# Impact of Total or Afferent Renal-Nerve Denervation on Cardiovascular and Renal Function in Sprague Dawley Rats Fed High-Fructose and High-Sodium Diet

**DOI:** 10.3390/nu18183067

**Published:** 2026-09-20

**Authors:** Sharif Hasan Siddiqui, Dragana Komnenov, Afoluso Olayonwa, Hainan Li, Jiemei Wang, Charles S. Chung, Ana M. Daugherty, Noreen F. Rossi

**Affiliations:** 1 Department of Physiology, School of Medicine, Wayne State University, 540 E. Canfield, Detroit, MI 48201, USA; hq4700@wayne.edu (S.H.S.); dkomneno@med.wayne.edu (D.K.); aolayonwa@wayne.edu (A.O.); cchung@wayne.edu (C.S.C.); 2 Department of Pharmaceutical Sciences, Eugene Applebaum Colledge of Pharmacy and Health Sciences, Wayne State University, 549 Mack Ave, Detroit, MI 48201, USA; fr3339@wayne.edu (H.L.); jiemei.wang@wayne.edu (J.W.); 3 Department of Psychology, Wayne State University, 87 Ferry St., Detroit, MI 48202, USA; dy6149@wayne.edu; 4 Institute of Gerontology, Wayne State University, 87 Ferry St., Detroit, MI 48202, USA

**Keywords:** albuminuria, arterial compliance, fructose, hypertension, renal sympathetic nerves

## Abstract

**Background:** Combined high fructose and high sodium diet (FHS) is linked to elevated blood pressure (BP), heightened sympathetic activity, and cardiovascular and renal dysfunction. Total-renal denervation is effective in lowering BP in some hypertension models. **Objectives:** The present studies tested whether selective afferent (A-RDNx) or total-renal denervation (T-RDNx) ameliorates FHS cardiovascular and renal function. **Methods:** Male and female Sprague Dawley rats were fed 20% glucose and 0.4% Na chow for one week then assigned to one of four groups: 20% glucose plus 0.4% Na sham-operation (G-Sham) or 20% fructose plus 4% Na with either sham-operation (F-Sham), A-RDNx, or T-RDNx for ~21 days. Telemetric BP and heart rate, echocardiography, pulse-wave velocity (PWV), and renal function were assessed. **Results:** T-RDNx reduced FHS-induced high BP in both sexes. LVEF did not differ, but global longitudinal strain showed hypercontractility in male F-Sham, which normalized with A-RDNx or T-RDNx. Increased LV perivascular fibrosis in F-Sham rats of both sexes was prevented by A-RDNx or T-RDNx. T-RDNx improved elevated PWV, endothelium-independent vasodilation by myography, and aortic oxidative stress in male F-Sham aortas. High plasma renin activity in male F-Sham decreased with T-RDNx to levels similar to G-Sham rats. Serum creatinine, urine albumin:creatinine ratio, and mesangial hypercellularity in F-Sham male rats decreased with A-RDNx or T-RDNx. Renal function was unchanged in female F-Sham; T-RDNx decreased albuminuria. **Conclusions:** Renal-sympathetic nerves play a crucial role in elevated BP, cardiac dysfunction, vascular stiffness, and renal impairment associated with FHS diet, particularly in male rats, and implicates a complex interplay with BP, the renin-angiotensin system, and oxidative stress.

## 1. Introduction

Hypertension is increasing worldwide [1] with a projected prevalence of 41.4% by 2030 in the United States [2] and 20.4% by 2040 globally [3]. As a leading risk factor for cardiovascular disease [4] or chronic kidney disease [5], hypertension ultimately leads to premature morbidity and mortality [3,6]. Several factors predispose to hypertension including age, race, genetic, and epigenetic background [7,8,9]; however, among the modifiable risk factors such as smoking, lack of exercise, and stress, the impact of a diet high in processed foods is also a strong contributor [10]. Combined high dietary fructose and sodium intake has been associated with increased blood pressure in both animals [11,12,13] and humans [14,15,16]. Although fructose consumption peaked in 1999, it remains far above the levels observed prior to 1970 when high fructose corn syrup was introduced. More-over, disparities in intake by socioeconomic status and ethnicity persist or have actually intensified [17,18]. Likewise, sodium intake also remains greater than the recommended guidelines, such that average intake is ~3.5 g/d [19] with values as high as 5–7 g/d when verified by 24-h urinary sodium excretion [20,21]. Although there has been some progress in decreasing fructose and salt intake, changing dietary habits remains challenging and intake remains high especially in some subgroups particularly young men [22]. While fructose or sodium each independently exert an effect on blood pressure, the combined impact of fructose and sodium ingestion on blood pressure and cardiorenal parameters appears to be at least additive, if not synergistic, via multiple mechanisms [23,24].

Preclinical studies indicate that populations consuming a combination of dietary high fructose and sodium further predisposes to urinary sodium retention, elevated blood pressures, an activated renin-angiotensin system (RAS), aortic stiffness, insulin resistance, and neuroexcitation [11,12,25,26,27]. Heightened sympathetic nervous system activity in rats on a 20% fructose plus 4% sodium (FHS) diet contributes to the failure to suppress renin secretion [28], sensitization of proximal renal tubular sodium reabsorption [13,29], and reduction in conduit artery compliance [30,31] despite both expanded extracellular volume and elevated blood pressure. Sympathoexcitation along with elevated angiotensin II (Ang II) and consequent oxidative stress thereby induces generalized vasoconstriction and endothelial dysfunction [32,33]. Renal cryo-denervation ameliorates blood pressure, plasma renin activity (PRA) and circulating Ang II levels [28,34]; acute pharmacologic sympathoinhibition improves aortic compliance in rats fed a high FHS diet [25].

Recent studies have shown that selective ablation of renal afferent nerves reduces blood pressure in the DOCA-salt rat [35] model of hypertension but not in others such as the Dahl S rat [36]. Clinical trials have demonstrated sustained efficacy of radiofrequency-based or ultrasound-based renal nerve denervation for treatment of resistant hypertension [37,38]. Importantly, a catheter-based method for afferent renal denervation in large animals has been developed that could be translated to clinical use for selected hypertensive individuals [39]. Since early-life ingestion of a high-FHS diet predisposes to salt-sensitive hypertension even in rats not genetically or hormonally predisposed to salt sensitivity, there is a gap in our preclinical knowledge as to whether selective afferent renal denervation vs. total renal denervation may have a beneficial impact on FHS hypertension and cardiovascular and renal parameters.

The present studies were designed to test the hypothesis that afferent renal denervation is as effective as total renal denervation in mitigating the rise in blood pressure, aortic stiffness, cardiac dysfunction, and the decline in renal function induced by a 3-week FHS diet in male and female Sprague Dawley rats.

## 2. Materials and Methods

### 2.1. Animal Protocol

All experiments were approved by the Institutional Animal Care and Use Committee (IACUC) of Wayne State University (23-08-6092) and adhered to the principles articulated in the *Guide for the Care and Use of Experimental Animals* (National Research Council 2011). The male and female 225 to 250 g Sprague Dawley (SD) rats were purchased from Inotiv Inc. (Indianapolis, IN, USA). The rats were allowed to acclimate to the vivarium with controlled light:dark cycle (lights on 0600-1800) and a temperature of 21–23 °C for a minimum of 4 days. They had ad libitum access to standard rat chow and water until they were entered into the investigational protocol.

**Figure 1:**
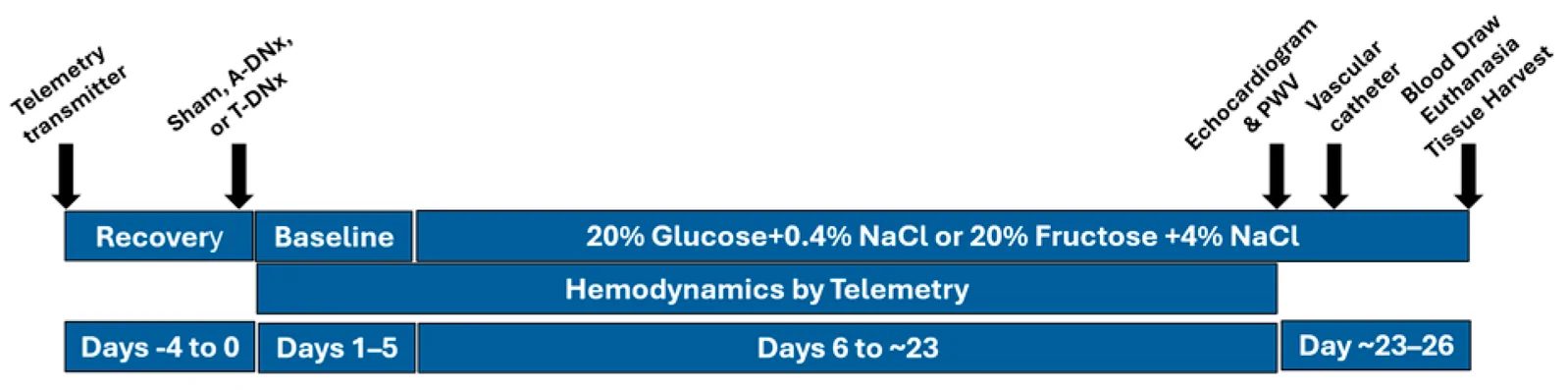
Schematic representation of study protocol and timeline.

The study design is illustrated in Figure 1. After arrival the rats were permitted to acclimate for ~4 days prior to surgical implantation of hemodynamic radio-transmitters and denervation procedures. Four days were allowed for recovery, then baseline arterial pressure and heart rate were recorded in their individual home cages for seven days during which they were provided with ad libitum chow containing 20% glucose plus 0.4% Na and water (TestDiet #1817915-209, Richmond, IN, USA). The rats were assigned to one of four groups: 20% glucose and 0.4% Na chow plus sham renal denervation (G-Sham), 20% fructose and 4% Na plus sham renal denervation (F-Sham), 20% fructose and 4% Na plus afferent renal nerve denervation (A-RDNx), or 20% fructose and 4% Na plus total renal nerve denervation (T-RDNx) (TestDiet #1818296-209, Richmond, IN, USA). Details regarding dietary composition are available in Supplemental Tables S1 and S2.

On day 22 ± 1, each of the rats underwent standard echocardiography, assessment of left ventricular (LV) strain, and aortic PWV while under 2.5% isoflurane anesthesia. The next day, a vascular catheter was inserted into the left carotid artery under ketamine/xylazine anesthesia. One to two days later, blood was collected via the catheter from the conscious rats that were then euthanized with sodium pentobarbital. Urine was obtained directly from the urinary bladder. Harvesting of heart and kidney tissue was performed for further analysis.

### 2.2. Surgical Procedures

Prior to each surgical procedure, analgesia was provided with Ethiqa 0.65 mg/kg s.c. (Fidelis, New Brunswick, NJ, USA).

Hemodynamic Telemetry Transmitter Placement. Transmitters were placed as previously described in our laboratory [11]. Rats were anesthetized with ketamine (80 mg/kg; Viatris, PA, USA) and xylazine 10 mg/kg (Dechra Veterinary Prod., Overland, KS, USA) i.p. The right femoral artery was exposed via an inguinal incision and the distal end was ligated with 3-0 suture. The proximal femoral artery was briefly occluded using a vascular clamp to permit the gel-filled catheter of the hemodynamic transmitter (HD-S10; Data Sciences International, New Brighton, MN, USA) to be inserted via a small arterial incision and advanced into the abdominal aorta. The catheter was secured with Prolene sutures and the transmitter body was tunneled subcutaneously and positioned dorsal to the right flank. The incision was closed using surgical clips.

Vascular Catheter Insertion. Under ketamine/xylazine anesthesia at the doses stated above, a midline incision was made in the ventral neck. A 22 g PFTE catheter was inserted into the left carotid artery and secured. The distal end of the catheter was tunneled subcutaneously and exteriorized at the base of the skull. The void volume of the catheter was filled with heparinized saline (1000 units/mL; Pfizer, Kalamazoo, MI, USA) and the catheter sealed. The incisions were closed using 4-0 Prolene suture (Ethicon; Johnson and Johnson, OH, USA), and the rats returned to their home cages to recover.

Renal Nerve Denervation Procedures. Rats were anesthetized with ketamine/xylazine. For total renal denervation (T-RDNx), the left and right kidneys were exposed via the retroperitoneal approach with left and right incisions. The renal nerve trunk was identified, surgically transected, and a 2–3 mm section of the nerve trunk was excised. The renal artery and vein were stripped of visible nerve bundles. The flank muscles were closed in layers and the incision with surgical clips. For afferent denervation (A-RDNx), the right kidney was exposed as above and the nerve was transected as in total renal denervation. The left kidney was exposed via the flank incision, the nerve trunk was identified and wrapped in gauze soaked with 33 mM capsaicin in normal saline for 15 min [35,40]. The gauze was removed and the flank muscles and surgical incision closed as for T-RDNx. Topical bupivacaine/lidocaine was applied along the incisions to prevent scratching. For sham procedures, the kidney and adnexa including the renal nerves were exposed bilaterally without transecting or soaking them. Rats were permitted to recover in their home cages.

### 2.3. Echocardiography

Echocardiograms were performed by an investigator blinded to the group assignment using Vevo 3100 (Visual Sonics, Bothell, WA, USA) following methods previously reported by our laboratory [12,25,41]. Briefly, after induction with 2.5% isoflurane, rats were maintained under 1.5% of isoflurane via an inhalation nose cone. The rats were kept recumbent on a heated stage to maintain body temperature at 37 °C monitored by a rectal probe. Limbs were affixed to the ECG electrodes with tape throughout the echocardiogram. All the images were acquired using an MX-250S transducer to obtain both the short and long axis following standard techniques. Left ventricular diastolic function was assessed using pulse wave Doppler recordings of transmitral flow velocities aligned in the apical four-chamber view. We analyzed the speckle tracking echocardiography using B-mode with the short and long axis. We used M-mode at the level of the cardiac papillary muscle with the short axis to assess systolic function and left ventricular dimensions.

Aortic pulse wave velocity (PWV) was measured using B-mode and pulse wave Doppler recordings at two positions along the aortic arch. The B-mode image was used to assess the distance between the two points which was then divided by the transit time using the ECG tracing as the reference.

### 2.4. Hormonal and Chemical Assays

Two to three days after echocardiography, blood glucose level was analyzed on ~50 µL whole blood obtained via the arterial catheter using a One-Touch Ultra Glucose monitor (LifeScan, Inc., Malvern, PA, USA) from the conscious rats in the morning (08:00–10:00). Then, 1.0 mL of blood was collected into prechilled tubes containing 50 µL ethylenediamine tetra-acetic acid and immediately centrifuged at 3000 rpm for 10 min at 4 °C. Plasma was stored at −70 °C for further analysis.

Insulin was determined using a rat insulin ELISA kit (Life Technologies-Invitrogen, Waltham, MA, USA). Plasma renin activity (PRA) was also measured by ELISA (Immuno-Biological Laboratories, Inc., Minneapolis, MN, USA). Urine albumin was assessed by ELISA (Rat Albumin ELISA Kit (Life Technologies-Invitrogen, Waltham, MA, USA). Plasma and urine creatinine levels were ascertained using a colorimetric assay kit (Cayman Chemical, Ann Arbor, MI, USA).

Confirmation of total denervation was accomplished by measuring renal tissue norepinephrine (NE) by high-performance liquid chromatography (HPLC) [28,42]. Frozen renal tissue was homogenized in 0.1 N perchloric acid with 1 mM EGTA and centrifuged at 4000 g for 10 min. The supernatant was diluted 1:10 in perchloric:EGTA and shipped on dry ice to the Vanderbilt Hormone Assay Core (https://www.vumc.org/hormone). The internal standard was dihydroxyl-benzylamine to monitor recovery and determine quantitation. Adequacy of total denervation was set a priori at an 85% decrease in renal tissue NE content vs. sham control.

### 2.5. Tissue Harvesting

Immediately after the blood draw, rats were euthanized with sodium pentobarbital 120 mg/kg i.v. Urine was collected directly from the bladder using a 22-gauge syringe and stored at −70 °C. The heart, aorta, and kidneys were harvested and kept in ice-cold saline. Kidneys and heart were weighed. Approximately 2 mm of the thoracic aorta was taken for myography and another 2 mm was embedded in OCT for evaluation of MitoSOX. Heart and kidney tissues were flash frozen and maintained at −70 °C; transverse tissue slices were placed in 10% formalin for subsequent histopathology and immunohistochemical analysis.

### 2.6. Myography

The aortic rings were immediately placed in the ice-cold physiological saline solution (PSS) containing (in mM) NaCl 118, NaHCO_3_ 24, KCl 4, glucose 5.6, MgSO_4_ 1, NaH_2_PO_4_ 0.435, and CaCl_2_ 1.8. Unwanted tissue and fat were removed as well as blood from the lumen. Then, a 2-mm segment of the aorta was mounted between the two wires of the myography chamber (DMT620M Danish MyoTechnology, Aarhus, Denmark) and permitted to equilibrate in PSS aerated with 95% O_2_ and 5% CO_2_ at 37 °C. Resting tension was adjusted and the rings were contracted with 120 mM KCl. Then, the target pressure was normalized and maximal contraction was accepted as evidence for viability. The rings were washed with PSS and dose-response curves were assessed from 1 nM to 0.1 mM with phenylephrine. Vasodilation was assessed after preconstricting with 0.1 mM phenylephrine and then applying 1 nM to 0.1 mM acetylcholine or sodium nitroprusside (SNP) [43].

### 2.7. Histology and Immunohistochemistry

Morphometry. Two-millimeter transverse slices of formalin-fixed heart and kidney were embedded and 10-µm sections were cut for further staining.

Morphometric analyses of heart and kidney were accomplished by a researcher blinded to group assignments. Cardiac tissue was stained with picrosirius red (Poylsciences, Inc., Warrington, PA, USA) and the percentage of cardiac fibrosis was assessed using ImageJ (version 1.54p). Kidney sections were stained with periodic acid Schiff (PAS) (Newcomer Supply, Middleton, WI, USA) [26]. Morphometry of glomerular mesangial cellularity was performed adhering to the parameters of the A.N.N. Working Group following the Oxford classification [44]. Forty glomeruli were analyzed per sample; left and right kidneys were analyzed separately. Mesangial cellularity was scored as follows: normal <4 mesangial cells/mesangial area, mild 4–5 mesangial cells/mesangial area, moderate 6–7 mesangial cells/mesangial area, and severe ≥8 mesangial cells/mesangial area.

CGRP immunohistochemistry. To assess adequacy of deafferentation, fixed kidney slices containing calyces and pelvis were deparaffinized, then incubated with polyclonal CGRP antibody at 1:500 dilution (Abcam; Cambridge, MA, USA), blocked with Peroxidaze 1 (Biocare Medical, Pacheco, CA, USA), followed by incubation with anti-rabbit:HRP. Slices were visualized, digitally photographed, and images evaluated for the presence/absence of CGRP by a researcher blinded to the experimental condition. Control tissues were run with each batch [45].

Mitochondrial superoxide staining. Thoracic aortic rings were immediately embedded in OCT compound (Tissue-Tek, Andwin Scientific, Simi Valley, CA, USA) in liquid nitrogen. Aortic sections (6 µm) were stained with 10 µM of dihydroethidium (DHE, D11347, Thermofisher, Waltham, MA, USA) for 13 min or with 5 µM of MitoSOXTM red mitochondrial superoxide indicator (M36008, Thermofisher, Waltham, MA, USA) for 10 min at 37 °C, gently washed three times with PBS, then mounted with DAPI Fluoromount-G (0100-20, SouthernBiotech, Birmingham, AL, USA). Fluorescent images were recorded by EVOS FL Imaging System (ThermoFisher Scientific, Waltham, MA, USA). Fluorescent threshold was set manually and kept consistent among all the sections [46].

### 2.8. Statistical Analysis

All data are reported as mean ± SE. Sample size was determined with PWV as the primary outcome of arterial stiffness resulting in six rats to achieve a power of 90% at an α-level of 0.05 to identify a difference of 100 mm/s in PWV with a standard deviation of 40 mm/s based on values from previous studies with this model [11,25]. Comparisons among experimental groups were accomplished by one-way ANOVA for multiple comparisons using Holm–Sidak post hoc analysis to control for false discovery rate, using GraphPad Prism, version 10.4.2.

Between-group differences in the pattern of change across the study design were tested in a repeated-measure ANOVA framework. This approach is consistent with hypothesized group effects in the average across days, and potentially also in the magnitude of change during a period of observation. The study was a 4 (between-subject, group assignment) × 2 (between-subject, sex) design with 18 days of observation for daytime measurements, and 17 days of observation for nighttime measurements. Outcomes of systolic and diastolic blood pressure and heart rate were tested separately. Prior to analysis, all data were screened and assumptions of multivariate normality, homogeneity of variance, and sphericity were assessed by standard practice. ANOVA is robust to minor deviations from normality; there was no evidence of multivariate outliers by Mahalanobis distance (critical χ^2^, α = 0.001), and all other assumptions were reasonably met, except sphericity (all Mauchly’s W, *p* < 0.01). Based on the average epsilon of all models below 0.75, a Greenhouse–Geiser correction to the within-subjects F-tests was used as a conservative adjustment that maintains power [47]. Type I error was controlled as a familywise error rate adjustment to the multiple models tested (omnibus F-test α′ = 0.01), and post hoc pairwise comparisons of group differences in estimated marginal means (EMMs) were made with Tukey adjustment. Analysis was completed with pairwise deletion of incomplete data in the outcome. Two animals in the A-RDNx group were missing at least one daytime measurement, resulting in *n* = 5 for that group in those analyses. Approximately half of all animals across treatment were missing at least one nighttime measurement, resulting in group sizes of G-Sham *n* = 5, F-Sham *n* = 9, A-RDNx *n* = 7, and T-RDNx *n* = 6. Data across the multivariate set were found to be missing not at random (Little’s χ^2^ (498) = 383312.68, *p* < 0.001); therefore, pairwise deletion was used to maximize statistical power; however, comparisons across daytime and nighttime effects should be made with this consideration.

With the available sample size, the test of the treatment and treatment × day effects had 80–95% power to detect moderate effect sizes (f = 0.17) of daytime effects and nighttime effects (f = 0.22–0.24) to statistical significance (α′ = 0.01), average correlation among measure = 0.5 [48].

## 3. Results

Initial body weights of all male and female rats were similar; however, male rats’ weights increased significantly in each of the groups except A-RDNx. Body weights of female rats increased but did not differ significantly from their initial values. As a result, the female body weight for each of the groups except A-RDNx was significantly lower than that of the corresponding male group (Table 1). The changes in body weight are depicted in Figure S1. Heart as well as left and right kidney weights of the female rats were significantly lower than those of the male rats from the same group except for the left A-RDNx kidney which was the kidney that underwent deafferentation only (Table 1). Blood glucose and plasma insulin were not available for all animals due to clotting of the catheter. The levels did not differ among any of the groups. The glucose:insulin ratio in the F-sham group of both male and female rats was modestly lower than other groups but not statistically significant (*p* = 0.398 males; *p* = 0.098 females; Table 2). Plasma renin activity (PRA) was significantly elevated in male F-Sham vs. G-Sham rats; T-RDNx prevented the increase in PRA. A-RDNx attenuated the increase in PRA but did not achieve significance (*p* = 0.08; Table 2).

### 3.1. Evaluation of Renal Deafferentation or Total Denervation

**Figure 2:**
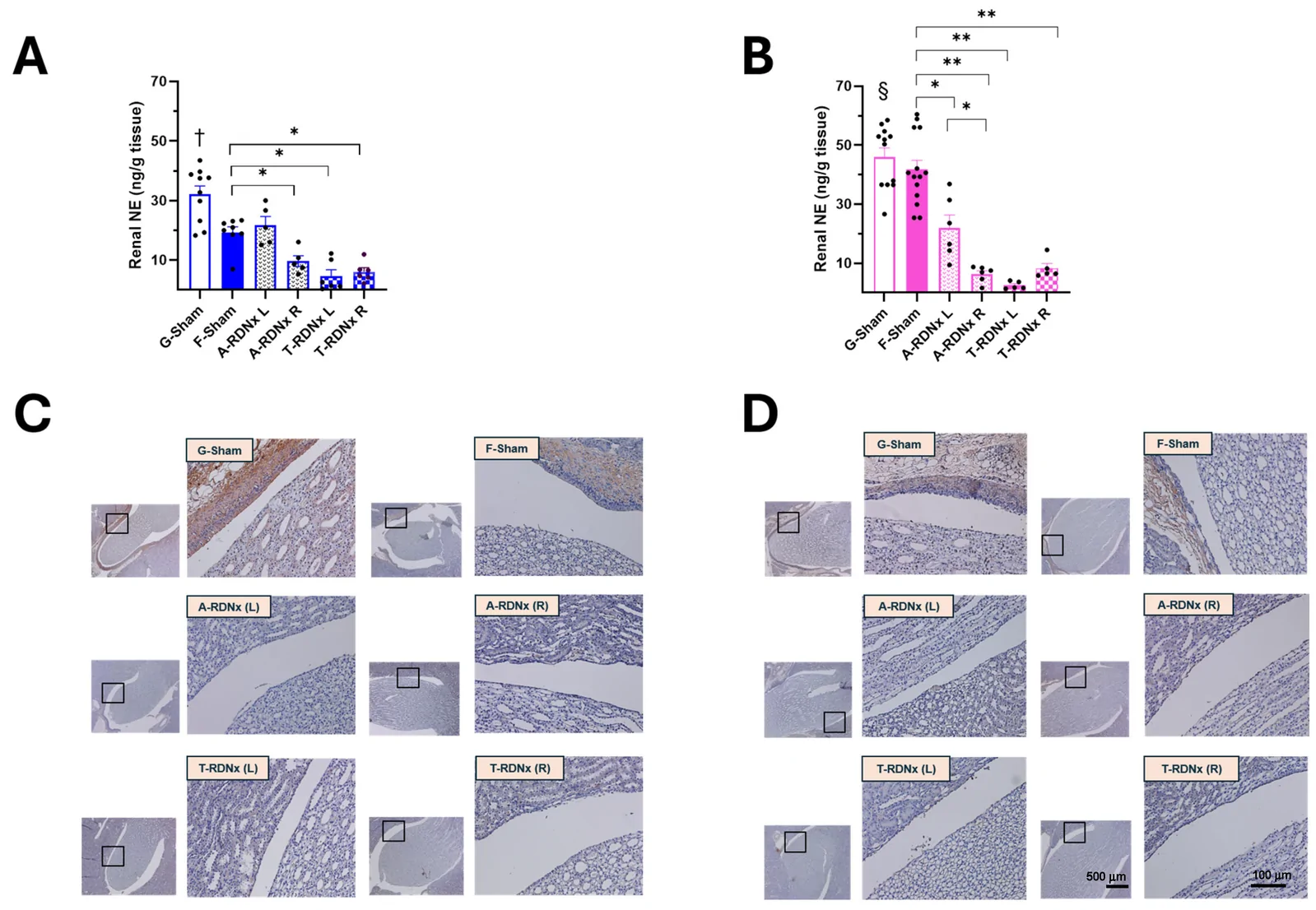
Renal norepinephrine (NE) content in male (**A**) and female (**B**) rats. Values for left and right kidneys of G-Sham and F-Sham were combined. Values are mean ± SE. * *p* < 0.01, ** *p* < 0.001 and indicated; † *p* < 0.01 vs. all other groups, § *p* < 0.001 vs. all groups except F-Sham by ANOVA. (**C**) Representative renal pelvicalyceal immuno-histochemical staining for CGRP in male rats. (**D**) Representative renal pelvicalyceal immuno-histochemical staining in female rats. G-Sham, 20% glucose + 0.4% Na; F-Sham, 20% fructose + 4% Na; A-RDNx, 20% fructose + 4% Na with afferent renal denervation; T-RDNx, 20% fructose + 4% Na with total renal denervation. Scale is 500 µm for 4× and 100 µm for 20× magnification.

G-Sham and F-sham kidney tissue from male rats had lower NE content than female G-Sham or F-sham rats respectively (*p* < 0.001 by two-way ANOVA). Male F-Sham rats exhibited lower renal NE than the male G-Sham group (Figure 2A). In male A-RDNx rats, the left kidney, which only underwent deafferentation with capsaicin-displayed NE content, similar to that of F-Sham male rats. The right kidney had significantly lower NE consistent with total denervation. In female rats (Figure 2B), NE content of the capsaicin-treated left kidney of A-RDNx was lower than F-Sham females although the right kidney that underwent total denervation displayed NE content significantly lower than either the L-ARDNx or F-Sham female kidneys. NE content was lower in both left and right kidneys of T-RDNX kidneys compared with either G-Sham or F-Sham groups of the same sex. Immunohistochemical staining of left and right kidneys of male (Figure 2C) and female rats (Figure 2D) depicts prominent staining for CGRP in the sham-denervated groups of both sexes. Deafferentation or total denervation eliminated CGRP immunoreactivity.

### 3.2. Impact on Cardiovascular Parameters

#### 3.2.1. Impact on Blood Pressure and Heart Rate

**Figure 3:**
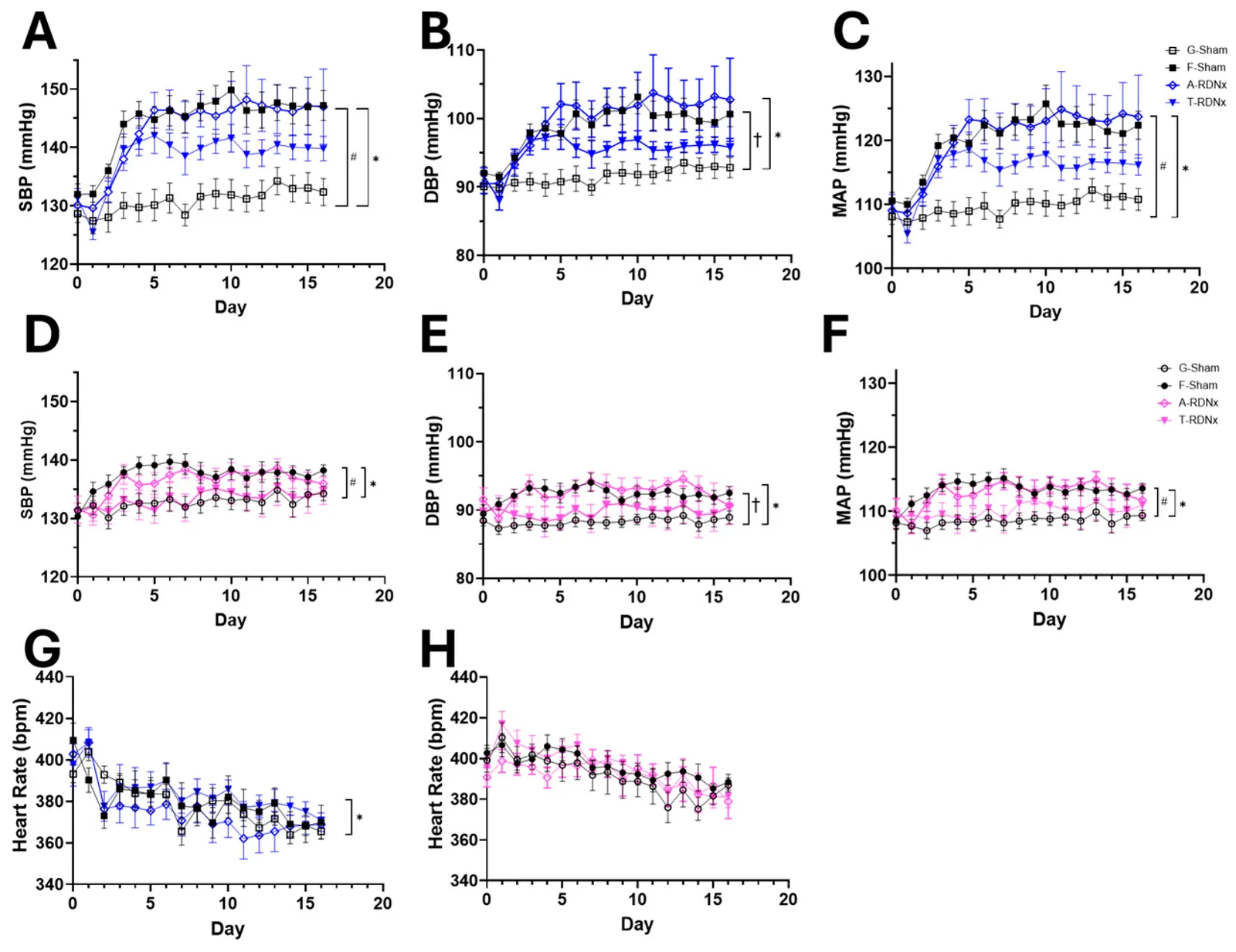
Daily 24-h average systolic, diastolic, and mean blood pressures and heart rate in male (**A**–**C**,**G**) and female (**D**–**F**,**H**) rat groups. SBP, systolic blood pressure; DBP, diastolic blood pressure; MAP, mean blood pressure; HR, heart rate; bpm, beats per minute. Symbols apply to all graphs; group names as in Figure 2. Values are mean ± SE; *n* values are as in Table 1. * *p* < 0.01 A-RDNx vs. G-Sham, ^#^ *p* < 0.02 F-Sham vs. G-Sham, ^†^ *p* < 0.05 F-Sham vs. G-Sham.

Blood pressure was measured from day 1 to the end of the study period. Figure 3 shows that average daily systolic and diastolic blood pressures were significantly higher in the F-Sham compared with the G-Sham groups in males (Figure 3A–C) and females (Figure 3D–F) respectively. Accordingly, MAP also differed. Importantly, A-RDNx did not alter the elevated pressures induced with FHS feeding. However, T-RDNx decreased blood pressures to levels comparable to G-Sham controls of the same sex. Average heart rate was lower in A-RDNx rats vs. G-Sham male rats; average heart rate did not differ among any of the female groups (Figure 3G,H). Sex did not modify the effect of treatment in any analysis (Day × Sex × Treatment, or Sex × Treatment; Table S3).

Figures S2 and S3 illustrate male and female daytime and nighttime blood pressure and heart rate, respectively. Detailed statistical analyses of treatment effects in daytime and nighttime blood pressure and heart rate as well as the impact of sex are provided in Table S3.

#### 3.2.2. Cardiac Parameters

Cardiac functional parameters assessed by M-mode echocardiography are depicted in Table 3. Representative M-mode images are provided in Figure S4. No significant changes were observed in any of the parameters among the groups of male rats. Specifically, systolic phenotypes such as LV ejection fraction, cardiac output, LV wall thicknesses, and mass did not differ. In addition, there was no evidence of diastolic dysfunction as measured by the ratio of early (E) to late (A) transmitral filing velocities. In female rats, there were no differences in any parameter between G-Sham and F-Sham groups. Notably, LV systolic wall thickness and LV mass were significantly lower in T-RDNx compared with F-Sham female rats. LV posterior wall thickness during diastole was greater in the A-RDNx group vs. either G-Sham or T-RDNx groups. Consistent with this finding, LV mass was significantly lower in the T-RDNx group compared with the A-RDNx female rats. Main effects testing showed sex had a significant effect (F = 5.05, *p* = 0.0005) and accounted for 46% of the variance whereas diet accounted for only 8.1% of the variance (F = 1.24, *p* = 0.310). Cardiac output did not differ among the female groups but the E/A ratio was higher in the A-RDNx group compared with either G-Sham or T-RDNx female rats. Diet accounted for 20.2% of the variance (F = 2.24, *p* = 0.072) and sex accounted for only 16.7% of the variance (F = 1.32, *p* = 0.269) by two-way ANOVA.

**Figure 4:**
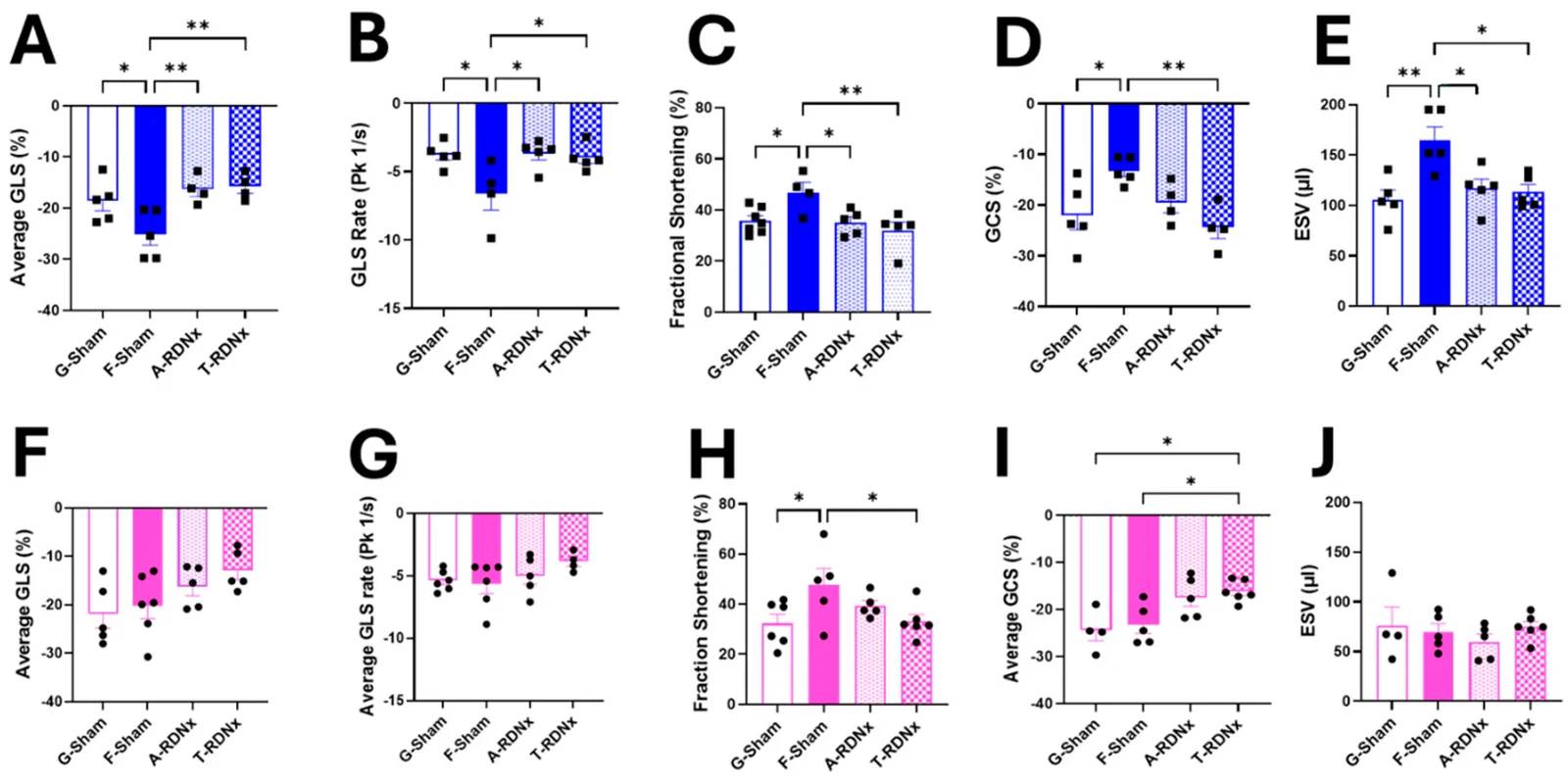
Average strain measurements for male (**A**–**E**) and female (**F**–**J**) groups by speckle-tracking echocardiography. Global longitudinal strain (GLS), GLS rate, fractional shortening, global circumferential strain (GCS and end systolic volume (ESV). Symbols apply to all graphs; group names as in Figure 2. Values are mean ± SE. * *p* < 0.05, ** *p* < 0.01.

Representative image analyses of speckle-tracking echocardiography are shown in Figure S4. In male rats, global longitudinal strain (GLS) and GLS strain rate was lower in F-Sham compared with G-Sham rats. GLS and GLS strain rate were improved by either A-RDNx or T-RDNx. (Figure 4A,B). In contrast, neither GLS nor GLS rate differed among any of the female groups (Figure 4F,G). For GLS, diet exerted a significant effect (F = 3.35, *p* = 0.032) but sex did not (F = 1.91, *p* = 0.128); however, sex accounted for 52% of the variance in GLS rate (F = 3.20, *p* = 0.020) but diet did not (F = 0.27, *p* = 0.895). Fractional shortening was significantly greater in F-Sham compared with G-Sham rats of both sexes (Figure 4C,H). Global circumferential strain (GCS) was lower in F-Sham vs. G-Sham male rats and was improved by T-RDNx (Figure 4D). In female rats, GCS did not differ in F-Sham vs. G-Sham rats, but was significantly lower than either group after T-RDNx (Figure 4I). Neither diet (F = 0.13, *p* = 0.971) nor sex (F = 1.94, *p* = 0.120) had an impact on the variances. Consistent with these measurements, end systolic volume (ESV) was higher in the F-Sham vs. G-Sham male group and was restored by either A-RDNx or T-RDNx (Figure 4E); ESV was similar across all female groups. Sex exerted a major effect on the variance in ESV (F = 12.01, *p* < 0.0001) whereas diet did not (F = 0.60, *p* = 0.667).

**Figure 5:**
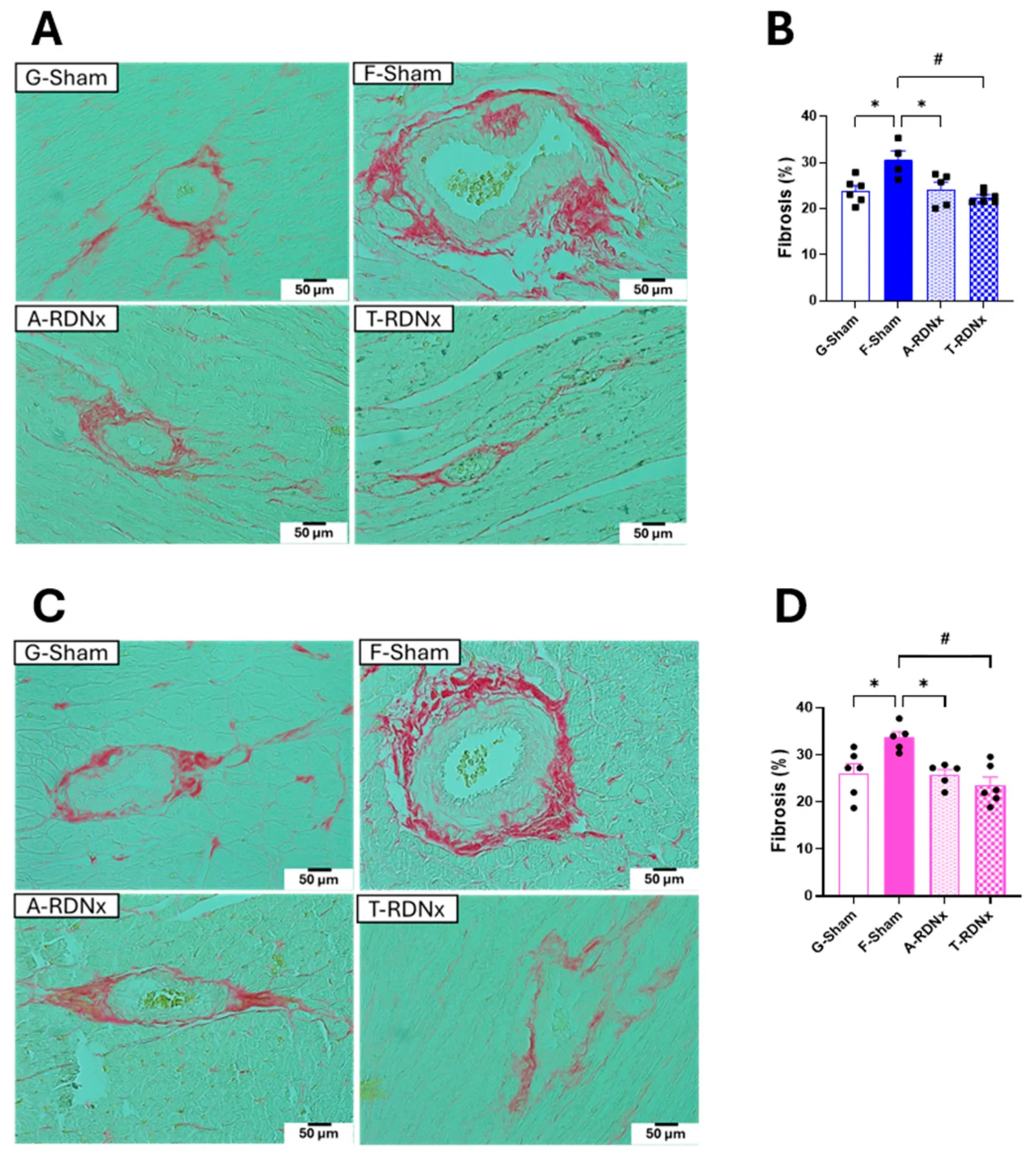
Histologic evaluation of cardiac fibrosis. Representative picrosirius red staining of left ventricle tissues in the four groups of male (**A**) and female (**C**) rats. Quantification of fibrotic area is shown in males (**B**) and females (**D**), respectively. Scale bar is 50 µm. Group names as in Figure 2. Values are mean ± SE. * *p* < 0.05, ^#^ *p* < 0.01.

LV fibrosis appeared to be localized primarily in the perivascular tissue rather than throughout the myocardium. Fibrosis was greater in F-Sham rats compared with G-Sham rats regardless of sex. LV fibrosis was significantly reduced in FHS fed rats in male and female rats with either A-RDNx or T-RDNx to a level comparable to the G-Sham group (Figure 5).

#### 3.2.3. Vascular Function In Vivo

**Figure 6:**
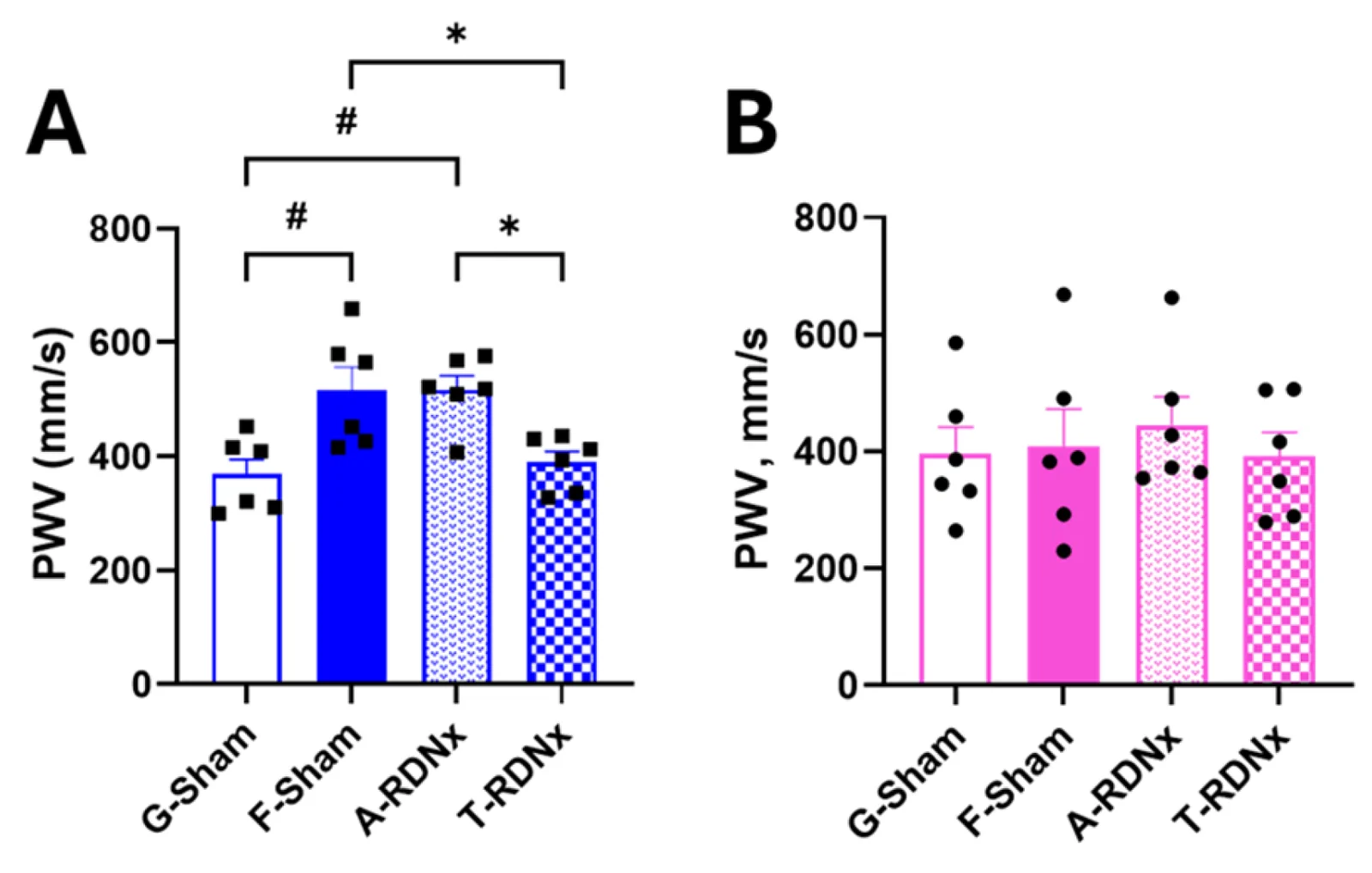
Aortic pulse wave velocity (PWV) in male (**A**) and female (**B**) rats. Group names as in Figure 2. Values are mean ± SE. * *p* < 0.05, ^#^ *p* < 0.01.

In male rats, aortic PWV was significantly elevated in F-Sham compared with G-Sham male rats. T-RDNx decreased PWV significantly vs. the F-Sham group to a level no different from the male G-Sham group. A-RDNx did not impact the effect of FHS feeding on aortic stiffness. PWV was comparable across all female groups (Figure 6). Neither diet (F = 0.54, *p* = 0.719) nor sex (F = 1.13, *p* = 0.372) accounted for the variances.

#### 3.2.4. Vascular Function Ex Vivo

**Figure 7:**
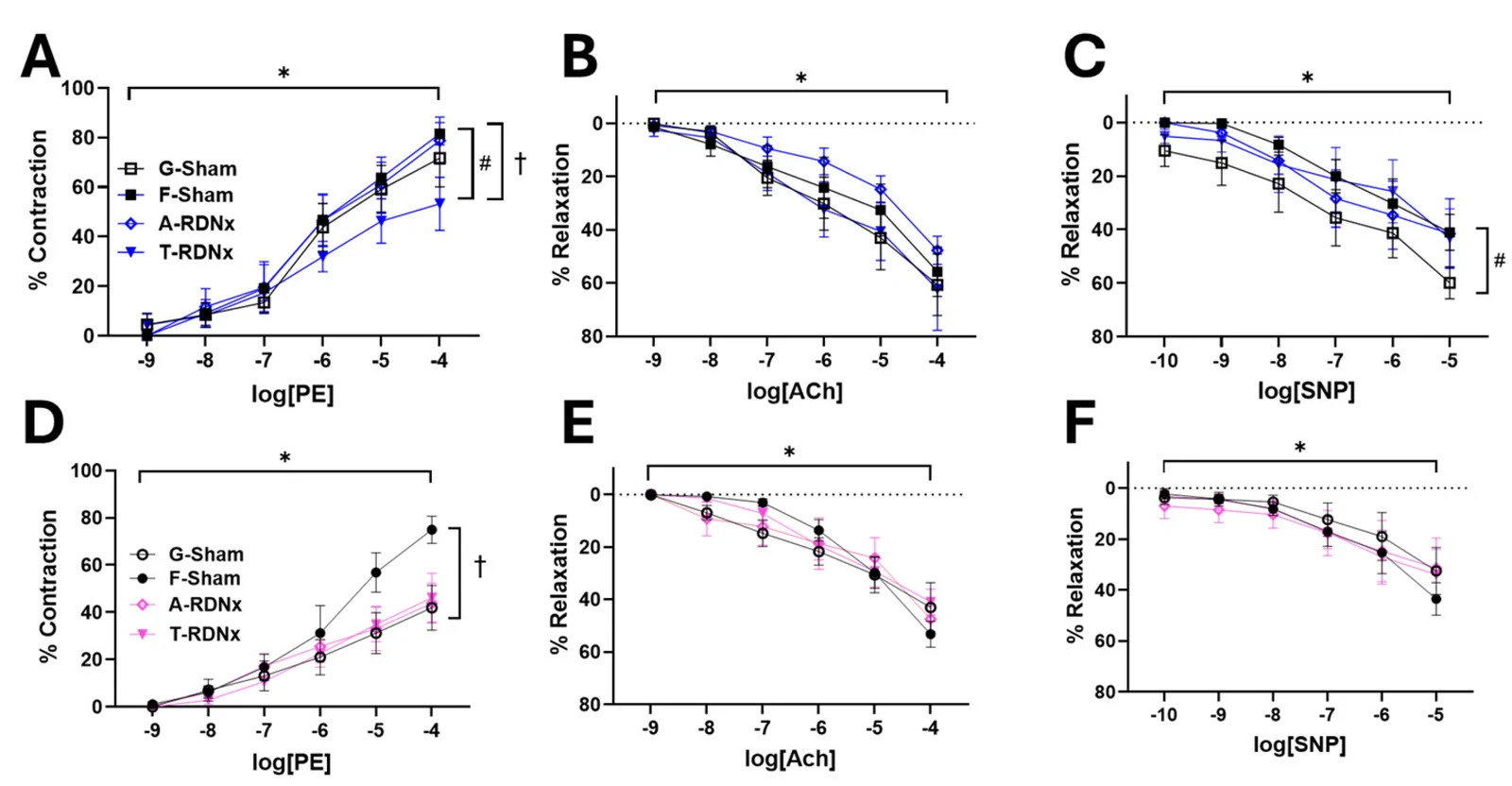
Ex vivo myography of thoracic aortic rings in male (**A**–**C**) and female (**D**–**F**) rats in response to phenylephrine (PE), acetylcholine (ACh), or sodium nitroprusside (SNP). Values are mean ± SE; *n* = 6 for all groups. * *p* < 0.0001 from baseline, ^†^ *p* < 0.006 vs. F-Sham, ^#^ *p* < 0.025 vs. G-Sham as shown by two-way ANOVA.

Results from ex vivo myography of thoracic rings taken at the level where PWV was assessed are shown in Figure 7. In male rats, phenylephrine induced dose-dependent vasoconstriction across all doses (*p* < 0.0001). Diet exerted no effect (*p* = 0.123, Figure 7A) but vasoconstriction was significantly less robust in T-RDNx compared with either G-Sham or F-Sham aortas. In female rats, phenylephrine also induced dose-dependent vasoconstriction (*p* < 0.0001), but G-Sham aortas displayed significantly less vasoconstriction than G-Sham male rat aortas (*p* < 0.0001). Female F-Sham aortas constricted comparable to the aortas from either G-Sham and F-Sham male rats and showed greater constriction than aortas from the other female groups (*p* < 0.006, Figure 7D). Acetylcholine produced vasodilation (*p* < 0.0001) that was similar in aortas from all male and female groups (Figure 7B,E). In contrast, vasodilation in response to SNP was impaired in male F-Sham vs. G-Sham aortas (*p* < 0.005) and neither A-RDNx nor T-RDNx improved the dilation. On the other hand, the response to SNP in all the female groups was similar (Figure 7C,F).

**Figure 8:**
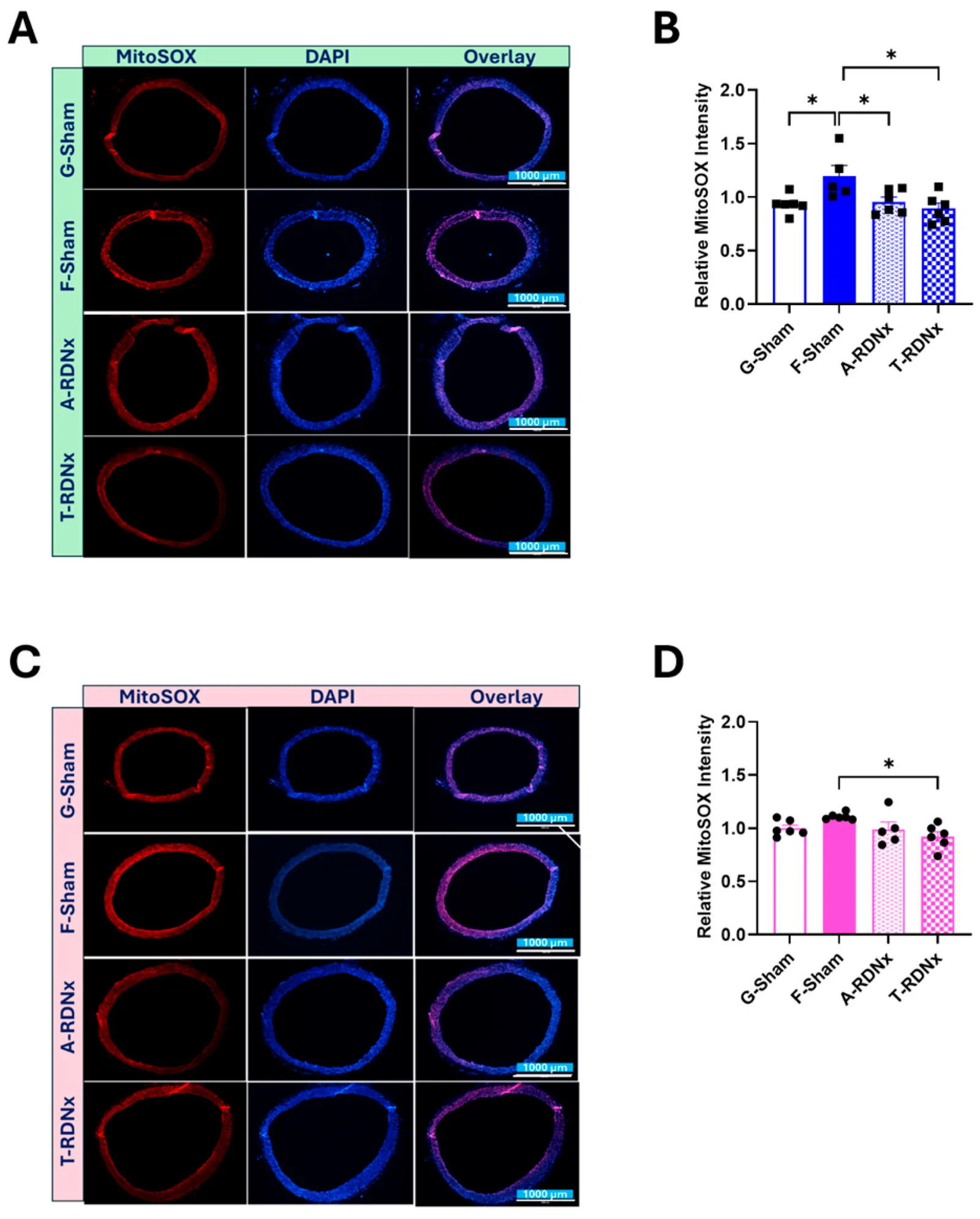
Aortic rings stained for superoxide with red mitochondrial superoxide indicator (MitoSOXTM), 4′6 diamidino-2-phenylindole (DAPI), and overlay from male (**A**) or female (**C**) rats in the four groups as indicated in Figure 2. Quantitation of immunofluorescence is shown in males (**B**) and females (**D**), respectively. Scale bar is 1000 µm. Values are mean ± SE. * *p* < 0.05.

Male F-Sham rats displayed greater mitochondrial oxidative stress than G-Sham male rats that was largely confined to the vascular smooth muscle of the medial layer; this effect was ameliorated by either A-RDNx or T-RDNx (*p* < 0.05 by one-way ANOVA). Although F-Sham female rats did not exhibit greater aortic oxidative stress, T-RDNx significantly attenuated MitoSOX staining (*p* < 0.05) (Figure 8).

### 3.3. Renal Parameters

#### 3.3.1. Glomerular Histology

**Figure 9:**
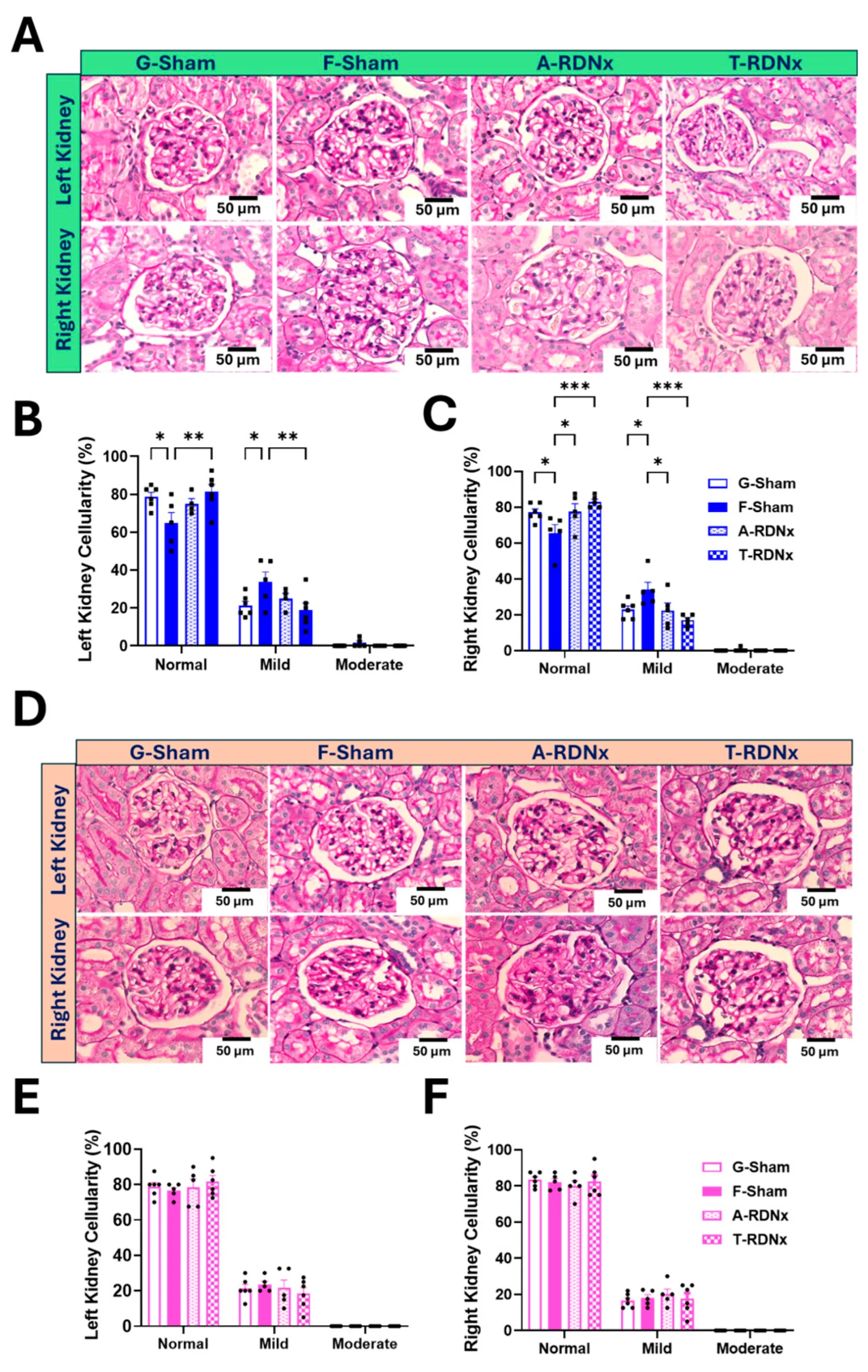
Periodic acid Schiff (PAS) staining of renal tissue depicting representative glomeruli from the four groups of male (**A**) and female (**D**) rats. Quantitative morphometric assessment for males (**B**,**C**) and females (**E**,**F**) shows percentage of glomeruli with mesangial hypercellularity: normal <4, mild 4–5, moderate 6–7 mesangial cells/area. No severe hypercellularity was noted. Number of observations indicate the number of kidneys evaluated; 40 glomeruli were evaluated blindly per kidney. * *p* < 0.05, ** *p* < 0.01, *** *p* < 0.001.

Histologic analysis of kidney tissues revealed a significantly greater proportion of glomeruli with mild mesangial hypercellularity as early as 3 weeks of FHS feeding in male rats that were ameliorated by T-RDNx but not A-RDNx in male rats (Figure 9A–C). Mild mesangial hypercellularity was similar across all female rat groups (Figure 9D–F).

#### 3.3.2. Renal Function

**Figure 10:**
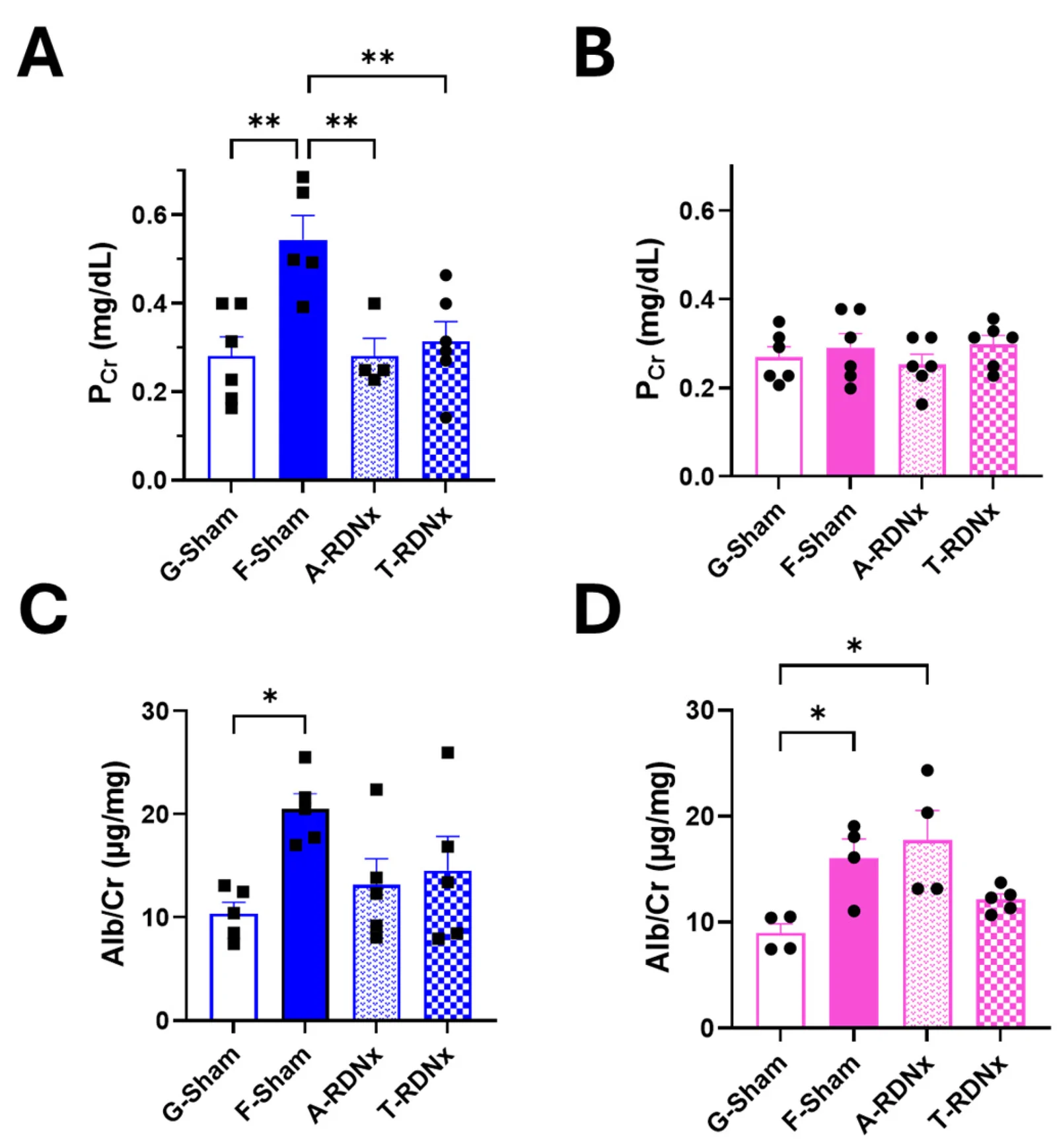
Renal function assessed as plasma creatinine (PCr; (**A**,**B**)) and urinary albumin to creatinine ratio (Alb/Cr; (**C**,**D**)) in the four groups of rats as in Figure 2. Values are mean ± SE. * *p* < 0.05, ** *p* < 0.01.

Serum creatinine was elevated in the F-Sham vs. G-Sham male rats but not in female rats. Either A-RDNx or T-RDNx prevented the rise in serum creatinine in the male groups (Figure 10A,B). Consistent with the decline in renal function and greater mesangial hypercellularity, F-Sham male rats also displayed significantly greater urine protein:urine creatinine ratio. Albuminuria was reduced by either A-RDNx and T-RDNx to levels no different from G-Sham group, but did not achieve significance vs. F-Sham male rats (Figure 10C). Although the female F-Sham group exhibited glomerular mesangial morphometry and renal function similar to female G-Sham female rats, albuminuria was significantly higher in the F-Sham and A-RDNx female groups vs. G-Sham. T-RDNx diminished albumin excretion but did not achieve statistical significance (Figure 10D).

## 4. Discussion

The present studies provide evidence for four major findings that address our initial hypothesis. First, blood pressure increases after three weeks of combined FHS diet in both males and female rats. Notably, the increase in males is more profound than in females. In contrast to total renal denervation, deafferentation of the renal nerves does not prevent the increase blood pressure. Second, although LVEF is not altered, global LV strain, which is a more sensitive index of early systolic dysfunction [49,50], is impaired in male rats on an FHS diet and is improved by either deafferentation or total renal denervation. Female rats do not exhibit changes in LV function although total denervation does improve global circumferential strain. These functional data are corroborated by histologic evidence of increased cardiac perivascular fibrosis which is ameliorated by both deafferentation and total denervation. Third, the greater aortic stiffness displayed by male but not female rats on the FHS diet is ameliorated only by total renal denervation. Ex vivo myography agrees with these observations since only total denervation results in attenuated vasoconstriction in response to phenylephrine in male rats even though mitochondrial superoxide generation in male aortas decreases with either renal afferent or total denervation. Interestingly, female rats on an FHS diet display a diminished vasoconstrictor response. Unlike female rats that maintain their ability to vasodilate, male rats have an impaired endothelium-independent vasodilatory response. Fourth, the decline in renal function indicated by the elevated plasma creatinine in male FHS-fed rats is consistent with direct measurements of lower glomerular filtration rate [11]. Deafferentation or total renal denervation normalized plasma creatinine. The early mesangial proliferation in the male rats is consistent with these renal functional responses. In contrast, female renal function and histology were unaffected by diet or denervation procedures. Although the increase in albuminuria is highly variable, the values are attenuated after total denervation to levels no different from rats fed glucose and a normal sodium diet. Taken as a whole, these results demonstrate a more nuanced contribution of renal nerves with an important contribution of renal effect sympathetic pathways to blood pressure regulation, renin release, and vascular dysfunction while renal afferent pathways contribute more selectively to cardiac and renal responses on an FHS diet.

The sympathetic nervous system plays a crucial role in several models of hypertension. For the most part, total renal denervation which ablates both efferent and afferent nerves decreases blood pressure in virtually every preclinical model of hypertension in rodents [51,52] and also in human hypertension [53,54]. The FHS diet results in an increase in blood pressure in both sexes, with a significant albeit lesser absolute magnitude in females. The present studies using a surgical approach to achieve total renal denervation confirm the reduction in blood pressure observed in male rats with cryo-denervation [28] and demonstrate that total denervation is also effective in female rats. Although stimulation of renal pelvic afferent nerves increases blood pressure [55], the role of afferent renal nerves in various hypertension models appears to be more selective. Renal deafferentation decreases blood pressure in the DOCA-salt [35], two kidney-one clip [56], or 5/6th nephrectomy models [57]. Renal deafferentation clearly does not ameliorate the rise in blood pressure seen with the FHS diet. This observation is in keeping with findings in the stroke-prone SHR rat [58], angiotensin II-salt hypertensive rat [59], the Dahl SS rat [40], or the autosomal recessive polycystic disease rat [60] that respond to total renal denervation but not to selective deafferentation. The mechanisms underpinning the effectiveness of deafferentation or lack thereof requires further inquiry.

The changes in cardiac function are more subtle. Although LVEF is not altered in either sex, the greater negativity of GLS and GLS strain rate suggest a possible hypercontractile state consistent with increased fractional shortening in the male rats fed an FHS diet. The increase in end systolic volume may be a result of less-effective contractility or due to the higher afterload in male rats. Notably, fibrosis is localized to perivascular regions rather than between cardiomyocytes may partially explain why LV contractility was not impaired, at least with this relatively short-term FHS diet. The only anomaly is the increase in fractional shortening that is seen in the female rats on the FHS diet that is not reflected in the other parameters. Both GLS and fractional shortening return to levels similar to those in glucose fed male rats after either afferent or total renal denervation. Afferent inputs from the kidney project from the spinal cord to brain regions regulating cardiac sympathetic activity [61,62,63]. The lower heart rate after renal deafferentation in male rats is consistent with interruption of renal afferent inputs. In addition, a role for RAS in cardiac fibrosis cannot be unequivocally excluded by these studies since PRA is elevated with FHS feeding in male rats and declines with total denervation. Even transient inhibition of RAS can induce sex-specific benefits in collagen and oxidative stress leading to cardio-protection and remodeling [64]. Thus, the diet may impact cardiac perfusion and contractility via the sympathetic nervous system or RAS alone or in combination.

Aortic stiffness assessed by PWV is distinctly increased in male but not female rats on the FHS diet. Notably, females may be protected by hormones that mitigate sympathetic outputs to the vasculature [65]. In contrast to cardiac function where either afferent or total denervation impart benefit, only total renal denervation improves aortic compliance [66,67]. This finding agrees with studies showing that acute systemic sympathoinhibition improves aortic compliance in male rats [25] and sympathomimetics decrease aortic compliance [68]. Sympathetic efferent nerves also stimulate renin secretion [69]. Collagen and elastin composition of the vascular wall or changes in cellular signaling in vascular smooth muscle are enhanced by RAS activity [70,71]. Thus, the contribution of an activated RAS cannot be unequivocally excluded in male rats since the elevated PRA in the FHS group is significantly inhibited by total denervation and would also be consistent with the lack of impact on PWV removal of afferent nerves which do not regulate PRA. The lack of changes in PRA may also, at least in part, be responsible for PWV not changing in female rats on the FHS diet. A role for estrogen receptor effects on neurovascular signal transduction cannot be excluded in the female rats.

In contrast to PWV that measures the structural rigidity of the aorta at a given point in time in vivo under one constellation of physiological parameters, myography assesses dynamic vascular reactivity over a range of selected agonists ex vivo. As such, female aortic constriction in response to α-adrenergic stimulation is lower than that of male aortas from glucose-fed rats. This is in keeping with earlier studies showing decreased vasoconstriction by females [72]. Notably, FHS feeding abolishes this difference and is ameliorated by total denervation in both sexes whereas deafferentation only benefited female rats.

Although endothelium-dependent vasodilation is unaffected by diet in either sex, impaired endothelial-independent vasodilation occurs only in male aortas and is not ameliorated by denervation consistent with studies showing that endothelial independent vasodilation is not affected by sympathetic inputs [73]. The heightened mitochondrial oxidative stress in aortas of the male rats has also been observed with aging [74]. The improvement in oxidative stress with denervation in males is consistent with the beneficial effect of antioxidant treatment on vascular function in male rats on FHS diet [75]. The present studies cannot distinguish whether the greater elevation in blood pressure in male rats that does not occur in female rats may independently influence vascular stiffness or reactivity or vice versa. The RAS system, specifically via angiotensin II, can regulate the activity of Nox2 and Nox 4 to produce reactive oxygen species [76]; it is not known whether there is sexual dimorphism in this response. Together these findings suggest a more complex interplay among blood pressure, sympathetic inputs, RAS, oxidative stress, and sex hormones on vascular compliance in this dietary model that bears further investigation.

After 3 weeks of the FHS diet, plasma creatinine nearly doubles in the male rats, which reflects a ~50% decrease in renal function. Urinary albumin:creatinine ratio is increased compared with male rats fed high glucose and normal sodium diet consistent with greater mesangial hypercellularity. These data align with an earlier study in which glomerular filtration rate was measured directly and decreased significantly along with an increase in urinary albumin excretion [11]. Deafferentation or total renal denervation decreased the albumin:creatinine ratio to levels comparable to that in glucose-fed control male rats. In male Dahl salt-sensitive rats fed high fructose for 12 weeks, a more profound rise in blood pressure and albuminuria were seen along with more advanced renal histopathology including glomerulosclerosis and interstitial fibrosis; however, creatinine clearance was unexpectedly higher in that group without a clear explanation [77]. In contrast, female rats fed the FHS diet display no change in renal function or histopathology compared with the glucose normal sodium diet. Regardless, the albumin:creatinine ratio is elevated and normalizes with only total renal denervation. It should be noted that the number of urinary albumin values in the females is limited due to technical issues limiting collection. Nonetheless, albuminuria is a very sensitive index of early renal damage and may precede histological changes in the female groups. In humans, albuminuria is a strong risk factor not only for progressive chronic kidney disease but also cardiovascular disease including major adverse cardiovascular events, heart failure, and mortality [78,79,80].

Substantial evidence exists from our own [28,75] and other laboratories [81,82,83,84] to show that 20% fructose imparts insulin resistance in rodent models, particularly in males. Although the glucose:insulin ratio as an index of insulin resistance in the FHS groups is not significantly lower in either sex in the present study due to insufficient power, either type of denervation approach increases this ratio suggesting a benefit to insulin sensitivity. Similarly, the effect on insulin sensitivity in human subjects who underwent renal denervation for other indications such as resistant hypertension is mixed due to limited observation periods, lack of randomization, and insufficient power [85,86,87].

### Limitations

The denervation procedures were performed at the initiation of dietary groupings rather than after establishment of elevated blood pressure as would generally be implemented in a therapeutic setting; however, the purpose of this study was to evaluate the potential mechanistic roles of afferent vs. efferent renal nerves with the FHS diet. Rather than perform bilateral deafferentation [40,59] or a right nephrectomy followed by deafferentation of the left kidney as in Banek et al. [35], we chose to perform total denervation of the right kidney and deafferentation of the left kidney. This present approach was equivalent to “neural absence” of the right kidney but avoided the confounder of that a single kidney would exert on assessment of the impact of diet on renal function due to reduced renal mass. In addition, it is unclear why renal norepinephrine content was 50% lower in the sham-denervated male high fructose-high sodium fed rats than in the glucose normal sodium-fed male rats, yet blood pressures were nonetheless significantly elevated. The male rats displayed weight gain and considerably greater visceral adiposity compared with the female rats which makes identifying nerves more challenging and may have resulted in some damage to efferent sympathetic nerve fibers in the sham rats. This was also our rationale in performing right total denervation rather than applying capsaicin bilaterally and risk inadequate application on the right side that is more difficult to access technically. Notably, renal norepinephrine content was not decreased in the sham-denervated high fructose and salt-fed female rats where the elevation in blood pressure was substantially less evident than in their male counterparts. Importantly, the present studies were performed well within the timeframe before reinnervation of the kidneys occurs [88]. Nonetheless, the findings support the contention that the effectiveness of total renal denervation requires at least a >70% reduction in tissue norepinephrine [89]. We acknowledge that the results with oxidative stress warrant future studies of NADPH oxidase pathway and its components. Likewise, identification of molecular targets involved in TGFβ and collagen signaling will provide mechanistic insights and possible targets for intervention in future studies. Sample size precluded a more extensive analysis of sex differences, and the tests reported are underpowered and so only large sex effects were supported and discussed. Based on the pattern of evidence and sexual dimorphisms noted in the literature, the impact of sex should be investigated more intentionally in the future.

## 5. Conclusions

The interactions of neural, cardiovascular and renal inputs on physiological functions that occur with a diet high in fructose and sodium are depicted. In summary, the present studies indicate that a diet high in both fructose and sodium exerts greater deleterious effects in male than in female rats. Systemic blood pressure is increased to a greater extent in males. Early LV dysfunction, aortic stiffness, renal impairment, and elevated renin secretion also occur in the male rats. Overall, total renal denervation ameliorates each of these parameters whereas selective renal deafferentation appears to benefit only aortic compliance and renal function. Thus, total renal denervation may impart benefits due to the direct effects on neuroexcitability and sympathetic inputs to cardiac, vascular, and renal tissues in this dietary model. The lower systemic blood pressure or suppressed renin-angiotensin system may also exert independent effects on the pathophysiology and ultimate end-organ function. The present study cannot determine whether the benefits of total renal denervation arise principally from lowering blood pressure or from additional blood pressure independent effects on sympathetic activity, renin release, or oxidative stress. A combination of these factors is quite likely. The more moderate increase in blood pressure in females but subtle changes in cardiac fibrosis and albuminuria are consistent with such an interpretation and suggest that studies are needed to identify the protective factors in the female sex. Limiting fructose and salt intake remains an important guideline for cardiovascular and renal health. These findings also strongly support a greater emphasis on examining the impact of sex as a biological variable with appropriate power a priori on distinct cardiovascular and renal parameters after renal denervation in clinical studies.

## Figures and Tables

**Table 1:** Initial and final body weights, heart, and kidney weights of rats in each group.

Group	*N*	Initial Body Weight (g)	Final Body Weight (g)	Heart Weight (g)	Kidney Weights	
					Left (g)	Right(g)
**Males**						
**G-Sham**	6	234 ± 7	311 ± 18 ^‡^	1.167 ± 0.034	1.076 ± 0.051	1.084 ± 0.035
**F-Sham**	6	235 ± 7	316 ± 12 ^‡^	1.080 ± 0.032	1.107 ± 0.075	1.057 ± 0.031
**A-RDNx**	6	251 ± 12	283 ± 16 *	1.059 ± 0.067	1.036 ± 0.053	1.038 ± 0.055
**T-RDNx**	6	248 ± 11	315 ± 8 ^‡^	1.099 ± 0.030	1.030 ± 0.020	1.044 ± 0.055
**Females**						
**G-Sham**	6	235 ± 8	246 ± 10 ^§^	0.889 ± 0.042 ^†^	0.745 ± 0.027 ^†^	0.756 ± 0.027 ^†^
**F-Sham**	8	232 ± 5	244 ± 4 ^§^	0.920 ± 0.030 ^†^	0.852 ± 0.026 ^†^	0.859 ± 0.036 ^†^
**A-RDNx**	6	239 ± 8	242 ± 8	0.955 ± 0.037	0.889 ± 0.038	0.886 ± 0.037 ^†^
**T-RDNx**	7	230 ± 12	226 ± 10 ^§^	0.887 ± 0.045 ^†^	0.843 ± 0.052 ^†^	0.849 ± 0.446 ^†^

Values are mean ± SE. * *p* < 0.05 vs. F-Sham same sex, ^†^ *p* < 0.05 vs. corresponding male group, ^§^ *p* < 0.001 vs. corresponding male group and ^‡^ *p* < 0.02 vs. initial body weight same sex.

**Table 2:** Glucose, glucose:insulin ratios and plasma renin activity in each rat group at harvest.

Group	*N*	Blood Glucose (mg/dL)	Insulin (µIU/mL)	Glucose:Insulin Ratio (nM/nM × 10^6^)	PRA
					(ng AngI/mL/h)
**Males**					
**G-Sham**	6	140 ± 15	17.4 ± 4.2	87 ± 14	0.100 ± 0.008
**F-Sham**	4	135 ± 5	19.4 ± 2.4	66 ± 9	0.201 ± 0.036 *
**A-RDNx**	5	154 ± 16	13.5 ± 1.6	97 ± 6	0.120 ± 0.023
**T-RDNx**	6	123 ± 9	16.3 ± 1.6	105 ± 23	0.099 ± 0.014 ^#^
**Females**					
**G-Sham**	6	130 ± 8	11.6 ± 1.7	116 ± 20	0.124 ± 0.019
**F-Sham**	5	130 ± 8	18.1 ± 2.5	67 ± 10	0.128 ± 0.015
**A-RDNx**	6	133 ± 10	16.5 ± 2.0	81 ± 11	0.120 ± 0.024
**T-RDNx**	6	150 ± 10	20.6 ± 3.7	75 ± 11	0.124 ± 0.020

PRA, plasma renin activity; Ang I, angiotensin I. Values are mean ± SE. * *p* < 0.05 vs. G-Sham; ^#^ *p* < 0.05 vs. F-Sham.

**Table 3:** M-mode echocardiographic parameters.

Males	G-Sham	F-Sham	A-RDNx	T-RDNx
***N***	6	6	6	6
**LVEF (%)**	81.1 ± 4.3	75.8 ± 3.75	73.5 ± 2.6	73.7 ± 5.5
**LVFS (%)**	52.0 ± 4.8	45.7 ± 3.4	43.0 ± 2.6	44.4 ± 5.0
**LVID_S_ (mm)**	2.7 ± 0.4	2.8 ± 0.34	2.7 ± 0.1	1.9 ± 0.4
**LVID_D_ (mm)**	5.5 ± 0.5	5.1 ± 0.3	4.8 ± 0.3	5.2 ± 0.3
**LVAW_S_ (mm)**	3.21 ± 0.10	3.31 ± 0.10	3.16 ± 0.25	3.16 ± 0.20
**LVAW_D_ (mm)**	2.26 ± 0.15	2.63 ± 0.19	2.30 ± 0.14	2.32 ± 0.14
**LVPW_S_ (mm)**	3.70 ± 0.10	3.48 ± 0.37	3.28.0 ± 0.37	2.94 ± 0.17
**LVPW_D_ (mm)**	2.58 ± 0.28	2.48 ± 0.22	2.40 ± 0.30	2.26 ± 0.12
**LV Mass (mg)**	781 ± 32	785 ± 93	734 ± 112	679 ± 74
**CO (mL/min)**	31.9 ± 4.0	32.6 ± 3.8	27.6 ± 4.3	31.0 ± 3.2
**E/A Ratio**	1.11 ± 0.13	1.15 ± 0.08	1.12 ± 0.08	1.08 ± 0.03
**Females**	**G-Sham**	**F-Sham**	**A-RDNx**	**T-RDNx**
***N***	6	6	6	6
**LVEF (%)**	75.7 ± 2.1	74.4 ± 3.3	68.3 ± 1.4	74.5 ± 3.1
**LVFS (%)**	45.2 ± 1.9	44.4 ± 3.3	38.4 ± 1.1	44.3 ± 2.9
**LVID_S_ (mm)**	2.9 ± 0.2	3.0 ± 0.3	3.2 ± 0.2	2.9 ± 0.2
**LVID_D_ (mm)**	5.4 ± 0.2	5.3 ± 0.4	5.2 ± 0.3	5.2 ± 0.2
**LVAW_S_ (mm)**	2.91 ± 0.08	3.14 ± 0.08	2.94 ± 0.14	2.73 ± 0.09 *
**LVAW_D_ (mm)**	2.13 ± 0.16	214 ± 0.07	2.11 ± 0.07	1.93 ± 0.08
**LVPW_S_ (mm)**	2.72 ± 0.17	2.93 ± 0.25	2.94 ± 0.17	2.79 ± 0.31
**LVPW_D_ (mm)**	1.55 ± 0.12	1.95 ± 0.18	2.05 ± 0.12 ^†^	1.59 ± 0.14 ^#^
**LV Mass (mg)**	491 ± 28	569 ± 18	529 ± 36	432 ± 30 *
**CO (mL/min)**	38.0 ± 3.1	33.5 ± 4.8	29.1 ± 3.9	32.7 ± 3.4
**E/A Ratio**	1.08 ± 0.02	1.17 ± 0.09	1.37 ± 0.12 ^†^	1.04 ± 0.06 ^#^

LVEF, left ventricular ejection fraction; LVFS, left ventricular fractional shortening; LVID_s_, left ventricular systolic internal diameter; LVID_D_, left ventricular diastolic internal diameter; LVAW, left ventricular anterior wall width; LVPW, left ventricular posterior wall width; LV Mass, left ventricular mass; CO, cardiac output; E/A Ratio, ratio of early to late transmitral filling velocities. Values are mean ± SE. * *p* < 0.05 vs. F-Sham, ^†^ *p* < 0.05 vs. G-Sham, ^#^ *p* < 0.05 vs. A-RDNx.

## Data Availability

Data generated or analyzed from this study are available upon reasonable request in compliance with the Department of Veterans Affairs data sharing policies from the corresponding author. The data are not publicly available per Department of Veterans Affairs policy.

## Supplementary Materials

The following supporting information can be downloaded at https://www.mdpi.com/article/10.3390/nu18183067/s1, Figure S1. Change in body weight from entry into protocol until day of harvest for male and female rats; Figure S2. Systolic (SBP), diastolic (DBP), mean (MAP) blood pressures and heart rate during 12 hr-day (light cycle 0600-1800; A,C,E,G) and 12-hr night (dark cycle 1800-0600; B,D,F,H) in male rats; Figure S3. Systolic (SBP), diastolic (DBP), mean (MAP) blood pressures and heart rate during 12 hr-day (light cycle 0600-1800; A,C,E,G) and 12-hr night (dark cycle 1800-0600; B,D,F,H) in female rats; Figure S4. Representative M-mode echocardiogram images in male (A) and female (B) rats from each of the groups; Figure S5. Representative strain measurements for male (A) and female (B) groups assessed by speckle tracking echocardiography; Table S1: TestDiet 20% Glucose, 1.1% K, 0.4% Na; Table S2: TestDiet 20% Fructose, 1.1% K, 4.0% Na; Table S3: Summary of Repeated Measures ANOVA Results.

## References

[1] Mills K.T., Stefanescu A., He J. (2020). The global epidemiology of hypertension. Nat. Rev. Nephrol..

[2] Heidenreich P.A., Trogdon J.G., Khavjou O.A., Butler J., Dracup K., Ezekowitz M.D., Finkelstein E.A., Hong Y., Johnston S.C., Khera A. (2011). Forecasting the future of cardiovascular disease in the United States: A policy statement from the American Heart Association. Circulation.

[3] Boateng E.B., Ampofo A.G. (2023). A glimpse into the future: Modelling global prevalence of hypertension. BMC Public Health.

[4] Rahimi K., Nazarzadeh M., Rodgers A. (2025). Advancing Risk-Based Approaches in Blood Pressure Management: Reflections on the 2025 AHA/ACC Statement. Hypertension.

[5] He Y., Tang W., Chen J., Tang J., Zheng Y., Wang X., Xing B., Li X., Xu Y., Wang X. (2025). Global burden of chronic kidney disease due to hypertension (1990–2021): A systematic analysis of epidemiological trends, risk factors, and projections to 2036 from the GBD 2021 study. BMC Nephrol..

[6] Roth G.A., Mensah G.A., Fuster V. (2020). The Global Burden of Cardiovascular Diseases and Risks: A Compass for Global Action. J. Am. Coll. Cardiol..

[7] Lackland D.T. (2014). Racial differences in hypertension: Implications for high blood pressure management. Am. J. Med. Sci..

[8] Silva A., Torres E.D.S., Santos do Nascimento M.D.V., Franco J.O., Amaral D.C., Corin A.S., Cavalcanti L.B., Maia M.B.S., de Souza Franco E. (2025). Genetic and Epigenetic Biomarkers in Hypertension Impact on the Effectiveness of Individualized Therapy: A Systematic Review. J. Cardiovasc. Pharmacol..

[9] McEniery C.M., Wilkinson I.B., Avolio A.P. (2007). Age, hypertension and arterial function. Clin. Exp. Pharmacol. Physiol..

[10] Scaranni P., Cardoso L.O., Chor D., Melo E.C.P., Matos S.M.A., Giatti L., Barreto S.M., da Fonseca M.J.M. (2021). Ultra-processed foods, changes in blood pressure and incidence of hypertension: The Brazilian Longitudinal Study of Adult Health (ELSA-Brasil). Public Health Nutr..

[11] Levanovich P.E., Daugherty A.M., Komnenov D., Rossi N.F. (2022). Dietary fructose and high salt in young male Sprague Dawley rats induces salt-sensitive changes in renal function in later life. Physiol. Rep..

[12] Levanovich P.E., Chung C.S., Komnenov D., Rossi N.F. (2021). Fructose plus High-Salt Diet in Early Life Results in Salt-Sensitive Cardiovascular Changes in Mature Male Sprague Dawley Rats. Nutrients.

[13] Gordish K.L., Kassem K.M., Ortiz P.A., Beierwaltes W.H. (2017). Moderate (20%) fructose-enriched diet stimulates salt-sensitive hypertension with increased salt retention and decreased renal nitric oxide. Physiol. Rep..

[14] Siddiqui S.H., Rossi N.F. (2024). Acute Intake of Fructose Increases Arterial Pressure in Humans: A Meta-Analysis and Systematic Review. Nutrients.

[15] Tappia P., Garriock E., Ramjiawan B., Moghadasian M. (2026). High fructose consumption induces cardiac dysfunction and vascular abnormalities. Can. J. Physiol. Pharmacol..

[16] McMillan R.K., Stock J.M., Romberger N.T., Wenner M.M., Chai S.C., Farquhar W.B. (2025). The impact of dietary sodium and fructose on renal sodium handling and blood pressure in healthy adults. Physiol. Rep..

[17] Dunford E.K., Miles D.R., Hollingsworth B.A., Heller S., Popkin B.M., Ng S.W., Taillie L.S. (2024). Defining “High-In” Saturated Fat, Sugar, and Sodium to Help Inform Front-of-Pack Labeling Efforts for Packaged Foods and Beverages in the United States. Nutrients.

[18] Vatavuk-Serrati G., Frank S.M., Ng S.W., Taillie L.S. (2024). Trends in Sugar from Packaged Foods and Beverages Purchased by US Households Between 2002 and 2020. J. Acad. Nutr. Diet..

[19] Brouillard A.M., Kraja A.T., Rich M.W. (2019). Trends in Dietary Sodium Intake in the United States and the Impact of USDA Guidelines: NHANES 1999–2016. Am. J. Med..

[20] Donfrancesco C., Lo Noce C., Russo O., Minutoli D., Di Lonardo A., Profumo E., Buttari B., Iacone R., Vespasiano F., Vannucchi S. (2021). Trend of salt intake measured by 24-h urine collection in the Italian adult population between the 2008 and 2018 CUORE project surveys. Nutr. Metab. Cardiovasc. Dis..

[21] Liu X., Bai Y., Li S., O’Donnell M., Mente A., Yin L., Hu B., Cheng X., Liu W., Bai X. (2021). Associations of estimated 24-h urinary sodium excretion with mortality and cardiovascular events in Chinese adults: A prospective cohort study. J. Hypertens..

[22] Chen Y., Wang J., Leonberg K.E., Chui K.K.H., Ausman L.M., Naumova E.N. (2025). Beyond the Average: Trends in Extreme Sodium Intake in the U.S. Population, 2003–2018. Nutrients.

[23] Soleimani M., Alborzi P. (2011). The role of salt in the pathogenesis of fructose-induced hypertension. Int. J. Nephrol..

[24] Hasbal N.B., Bakir C.N., Incir S., Siriopol D., Sanchez-Lozada L.G., Lanaspa M.A., Johnson R.J., Kanbay M. (2024). A study on the early metabolic effects of salt and fructose consumption: The protective role of water. Hypertens. Res..

[25] Komnenov D., Rossi N.F. (2023). Fructose-induced salt-sensitive blood pressure differentially affects sympathetically mediated aortic stiffness in male and female Sprague-Dawley rats. Physiol. Rep..

[26] Siddiqui S.H., Pitpitan R., Boychev B., Komnenov D., Rossi N.F. (2024). Impact of inhibition of the renin-angiotensin system on early cardiac and renal abnormalities in Sprague Dawley rats fed short-term high fructose plus high salt diet. Front. Nutr..

[27] Gonzalez-Vicente A., Hong N.J., Yang N., Cabral P.D., Berthiaume J.M., Dominici F.P., Garvin J.L. (2018). Dietary Fructose Increases the Sensitivity of Proximal Tubules to Angiotensin II in Rats Fed High-Salt Diets. Nutrients.

[28] Soncrant T., Komnenov D., Beierwaltes W.H., Chen H., Wu M., Rossi N.F. (2018). Bilateral renal cryodenervation decreases arterial pressure and improves insulin sensitivity in fructose-fed Sprague-Dawley rats. Am. J. Physiol.-Regul. Integr. Comp. Physiol..

[29] Cabral P.D., Hong N.J., Hye Khan M.A., Ortiz P.A., Beierwaltes W.H., Imig J.D., Garvin J.L. (2014). Fructose stimulates Na/H exchange activity and sensitizes the proximal tubule to angiotensin II. Hypertension.

[30] Harvey R.E., Barnes J.N., Hart E.C., Nicholson W.T., Joyner M.J., Casey D.P. (2017). Influence of sympathetic nerve activity on aortic hemodynamics and pulse wave velocity in women. Am. J. Physiol.-Heart Circ. Physiol..

[31] Kalfon R., Campbell J., Alvarez-Alvarado S., Figueroa A. (2015). Aortic Hemodynamics and Arterial Stiffness Responses to Muscle Metaboreflex Activation with Concurrent Cold Pressor Test. Am. J. Hypertens..

[32] Klein A.V., Kiat H. (2015). The mechanisms underlying fructose-induced hypertension: A review. J. Hypertens..

[33] Münzel T., Camici G.G., Maack C., Bonetti N.R., Fuster V., Kovacic J.C. (2017). Impact of Oxidative Stress on the Heart and Vasculature: Part 2 of a 3-Part Series. J. Am. Coll. Cardiol..

[34] Mahfoud F., Townsend R.R., Kandzari D.E., Kario K., Schmieder R.E., Tsioufis K., Pocock S., David S., Patel K., Rao A. (2021). Changes in Plasma Renin Activity After Renal Artery Sympathetic Denervation. J. Am. Coll. Cardiol..

[35] Banek C.T., Gauthier M.M., Baumann D.C., Van Helden D., Asirvatham-Jeyaraj N., Panoskaltsis-Mortari A., Fink G.D., Osborn J.W. (2018). Targeted afferent renal denervation reduces arterial pressure but not renal inflammation in established DOCA-salt hypertension in the rat. Am. J. Physiol.-Regul. Integr. Comp. Physiol..

[36] Foss J.D., Fink G.D., Osborn J.W. (2016). Differential role of afferent and efferent renal nerves in the maintenance of early- and late-phase Dahl S hypertension. Am. J. Physiol.-Regul. Integr. Comp. Physiol..

[37] Kandzari D.E., Mahfoud F., Townsend R.R., Kario K., Weber M.A., Schmieder R.E., Tsioufis K., Pocock S., Liu M., DeBruin V. (2025). Long-Term Safety and Efficacy of Renal Denervation: 24-Month Results From the SPYRAL HTN-ON MED Trial. Circ. Cardiovasc. Interv..

[38] Bloch M.J., Kirtane A.J., Azizi M., Mahfoud F., Basile J., Daemen J., Saxena M., Thackeray L., McGuire M., Claude L. (2024). 36-month durability of ultrasound renal denervation for hypertension resistant to combination therapy in RADIANCE-HTN TRIO. Hypertens. Res..

[39] de la Cruz-Lynch A., Dailey-Krempel B., Dayton A., Nguyen D.T., Tyshynsky R., Van Helden D., Lahti M., Carney J., Evans L., Vulchanova L. (2025). A Novel Catheter-Based Method for Denervation of Afferent Renal Nerves in Sheep. Cardiovasc. Eng. Technol..

[40] Frame A.A., Carmichael C.Y., Kuwabara J.T., Cunningham J.T., Wainford R.D. (2019). Role of the afferent renal nerves in sodium homeostasis and blood pressure regulation in rats. Exp. Physiol..

[41] Siddiqui S.H., Komnenov D., Olayonwa A., Rossi N.F. (2026). Acute V(1b) vasopressin receptor antagonism ameliorates cardiovascular parameters in ovariectomized female rats subjected to chronic mild unpredictable stress. J. Neuroendocrinol..

[42] Rossi N.F., Pajewski R., Chen H., Littrup P.J., Maliszewska-Scislo M., Zenner Z., Rishi A.K., Levi E., Soncrant T., Komnenov D. (2016). Hemodynamic and neural responses to renal denervation of the nerve to the clipped kidney by cryoablation in two-kidney, one-clip hypertensive rats. Am. J. Physiol.-Regul. Integr. Comp. Physiol..

[43] Rastogi S., Grimm A., Biby B., Mathieu L., Trinh B., Nunes K.P. (2025). Chronic Treatment with Curcumin Prevents Vascular Dysfunction in the Aorta of Type 1 Diabetes by Restoring Ca^2+^ Mishandling and Modulating HSP70 Levels. Cells.

[44] Cattran D.C., Coppo R., Cook H.T., Feehally J., Roberts I.S., Troyanov S., Alpers C.E., Amore A., Barratt J., A Working Group of the International IgA Nephropathy Network and the Renal Pathology Society (2009). The Oxford classification of IgA nephropathy: Rationale, clinicopathological correlations, and classification. Kidney Int..

[45] Foss J.D., Wainford R.D., Engeland W.C., Fink G.D., Osborn J.W. (2015). A novel method of selective ablation of afferent renal nerves by periaxonal application of capsaicin. Am. J. Physiol.-Regul. Integr. Comp. Physiol..

[46] Li H., Nguyen H., Meda Venkata S.P., Koh J.Y., Kowluru A., Li L., Rossi N.F., Chen W., Wang J.M. (2021). Novel Role of GPR35 (G-Protein-Coupled Receptor 35) in the Regulation of Endothelial Cell Function and Blood Pressure. Hypertension.

[47] Tabachnick B.G., Fidell L.S. (2007). Using Multivariate Statistics.

[48] Faul F., Erdfelder E., Buchner A., Lang A.G. (2009). Statistical power analyses using G*Power 3.1: Tests for correlation and regression analyses. Behav. Res. Methods.

[49] Potter E., Marwick T.H. (2018). Assessment of Left Ventricular Function by Echocardiography: The Case for Routinely Adding Global Longitudinal Strain to Ejection Fraction. JACC Cardiovasc. Imaging.

[50] Mihos C.G., Liu J.E., Anderson K.M., Pernetz M.A., O’Driscoll J.M., Aurigemma G.P., Ujueta F., Wessly P., American Heart Association Council on Peripheral Vascular Disease, Council on Cardiovascular and Stroke Nursing (2025). Speckle-Tracking Strain Echocardiography for the Assessment of Left Ventricular Structure and Function: A Scientific Statement From the American Heart Association. Circulation.

[51] Osborn J.W., Foss J.D. (2017). Renal Nerves and Long-Term Control of Arterial Pressure. Compr. Physiol..

[52] Evans L.C., Dayton A., Osborn J.W. (2025). Renal nerves in physiology, pathophysiology and interoception. Nat. Rev. Nephrol..

[53] Katsurada K., Shinohara K., Aoki J., Nanto S., Kario K. (2022). Renal denervation: Basic and clinical evidence. Hypertens. Res..

[54] Fisher N.D.L., Kirtane A.J. (2025). Renal denervation for hypertension. Nat. Rev. Cardiol..

[55] Patel K.P., Knuepfer M.M. (1986). Effect of afferent renal nerve stimulation on blood pressure, heart rate and noradrenergic activity in conscious rats. J. Auton. Nerv. Syst..

[56] Lopes N.R., Milanez M.I.O., Martins B.S., Veiga A.C., Ferreira G.R., Gomes G.N., Girardi A.C., Carvalho P.M., Nogueira F.N., Campos R.R. (2020). Afferent innervation of the ischemic kidney contributes to renal dysfunction in renovascular hypertensive rats. Pflug. Arch.-Eur. J. Physiol..

[57] Campese V.M., Kogosov E. (1995). Renal afferent denervation prevents hypertension in rats with chronic renal failure. Hypertension.

[58] Ikeda S., Shinohara K., Kashihara S., Matsumoto S., Yoshida D., Nakashima R., Ono Y., Nishihara M., Katsurada K., Tsutsui H. (2023). Contribution of afferent renal nerve signals to acute and chronic blood pressure regulation in stroke-prone spontaneously hypertensive rats. Hypertens. Res..

[59] Foss J.D., Fiege J., Shimizu Y., Collister J.P., Mayerhofer T., Wood L., Osborn J.W. (2018). Role of afferent and efferent renal nerves in the development of AngII-salt hypertension in rats. Physiol. Rep..

[60] Gauthier M.M., Dennis M.R., Morales M.N., Brooks H.L., Banek C.T. (2022). Contributions of afferent and sympathetic renal nerves to cystogenesis and arterial pressure regulation in a preclinical model of autosomal recessive polycystic kidney disease. Am. J. Physiol.-Ren. Physiol..

[61] Ciriello J., Calaresu F.R. (1983). Central projections of afferent renal fibers in the rat: An anterograde transport study of horseradish peroxidase. J. Auton. Nerv. Syst..

[62] Xu B., Zheng H., Liu X., Patel K.P. (2015). Activation of afferent renal nerves modulates RVLM-projecting PVN neurons. Am. J. Physiol.-Heart Circ. Physiol..

[63] Huang B., Yu L., Scherlag B.J., Wang S., He B., Yang K., Liao K., Lu Z., He W., Zhang L. (2014). Left renal nerves stimulation facilitates ischemia-induced ventricular arrhythmia by increasing nerve activity of left stellate ganglion. J. Cardiovasc. Electrophysiol..

[64] Garvin A.M., Floyd D.B., Bailey A.C., Lindsey M.L., Carroll C.C., Hale T.M. (2025). Transient angiotensin-converting enzyme inhibition confers sex-specific protection against angiotensin II-induced cardiac remodeling. Am. J. Physiol.-Cell Physiol..

[65] Saleh M.C., Connell B.J., Saleh T.M. (2000). Autonomic and cardiovascular reflex responses to central estrogen injection in ovariectomized female rats. Brain Res..

[66] Han Y., Li X., Zhou S., Meng G., Xiao Y., Zhang W., Wang Z., Xie L., Liu Z., Lu H. (2012). 17ß-estradiol antagonizes the down-regulation of ERα/NOS-3 signaling in vascular endothelial dysfunction of female diabetic rats. PLoS ONE.

[67] Zhu Y., Bian Z., Lu P., Karas R.H., Bao L., Cox D., Hodgin J., Shaul P.W., Thoren P., Smithies O. (2002). Abnormal vascular function and hypertension in mice deficient in estrogen receptor β. Science.

[68] Alicandri C.L., Fariello R., Agabiti-Rosei E., Romanelli G., Muiesan G. (1980). Influence of the sympathetic nervous system on aortic compliance. Clin. Sci..

[69] Kopp U.C., DiBona G.F. (1993). Neural regulation of renin secretion. Semin. Nephrol..

[70] Han W.Q., Wu L.Y., Zhou H.Y., Zhang J., Che Z.Q., Wu Y.J., Liu J.J., Zhu D.L., Gao P.J. (2009). Changes in the composition of the thoracic aortic wall in spontaneously hypertensive rats treated with losartan or spironolactone. Clin. Exp. Pharmacol. Physiol..

[71] Rastogi S., Liaw J., Zhai Y., Karpova T., Gu L., Nunes K. (2025). Effect of Antihypertensive Losartan on Ca^2+^ Mobilization in the Aorta of Middle-Aged Spontaneously Hypertensive Female Rats. J. Cardiovasc. Dev. Dis..

[72] de Oliveira A.A., Priviero F., Delgado A., Dong P., Mendoza V.O., Gu L., Webb R.C., Nunes K.P. (2022). Connecting Aortic Stiffness to Vascular Contraction: Does Sex Matter?. Int. J. Mol. Sci..

[73] Lamb I.R., Novielli N.M. (2017). Characterizing the sympatholytic role of endothelial-dependent vasodilator signalling during handgrip exercise. J. Physiol..

[74] Zhou R.H., Vendrov A.E., Tchivilev I., Niu X.L., Molnar K.C., Rojas M., Carter J.D., Tong H., Stouffer G.A., Madamanchi N.R. (2012). Mitochondrial oxidative stress in aortic stiffening with age: The role of smooth muscle cell function. Arter. Thromb. Vasc. Biol..

[75] Komnenov D., Levanovich P.E., Perecki N., Chung C.S., Rossi N.F. (2020). Aortic Stiffness and Diastolic Dysfunction in Sprague Dawley Rats Consuming Short-Term Fructose Plus High Salt Diet. Integr. Blood Press. Control.

[76] Montezano A.C., Touyz R.M. (2014). Reactive oxygen species, vascular Noxs, and hypertension: Focus on translational and clinical research. Antioxid. Redox Signal..

[77] Xu L., Hu G., Qiu J., Fan Y., Ma Y., Miura T., Kohzuki M., Ito O. (2021). High Fructose-Induced Hypertension and Renal Damage Are Exaggerated in Dahl Salt-Sensitive Rats via Renal Renin-Angiotensin System Activation. J. Am. Heart Assoc..

[78] Ninomiya T., Perkovic V., de Galan B.E., Zoungas S., Pillai A., Jardine M., Patel A., Cass A., Neal B., Poulter N. (2009). Albuminuria and kidney function independently predict cardiovascular and renal outcomes in diabetes. J. Am. Soc. Nephrol. JASN.

[79] Barzilay J.I., Farag Y.M.K., Durthaler J. (2024). Albuminuria: An Underappreciated Risk Factor for Cardiovascular Disease. J. Am. Heart Assoc..

[80] Yaqoob K., Naderi H., Thomson R.J., Aksentijevic D., Jensen M.T., Munroe P.B., Petersen S.E., Aung N., Yaqoob M.M. (2025). Prognostic impact of albuminuria in early-stage chronic kidney disease on cardiovascular outcomes: A cohort study. Heart.

[81] Dupas J., Goanvec C., Feray A., Guernec A., Alain C., Guerrero F., Mansourati J. (2016). Progressive Induction of Type 2 Diabetes: Effects of a Reality-Like Fructose Enriched Diet in Young Wistar Rats. PLoS ONE.

[82] Stranahan A.M., Norman E.D., Lee K., Cutler R.G., Telljohann R.S., Egan J.M., Mattson M.P. (2008). Diet-induced insulin resistance impairs hippocampal synaptic plasticity and cognition in middle-aged rats. Hippocampus.

[83] Dupas J., Feray A., Goanvec C., Guernec A., Samson N., Bougaran P., Guerrero F., Mansourati J. (2017). Metabolic Syndrome and Hypertension Resulting from Fructose Enriched Diet in Wistar Rats. BioMed Res. Int..

[84] Mamikutty N., Thent Z.C., Sapri S.R., Sahruddin N.N., Mohd Yusof M.R., Haji Suhaimi F. (2014). The establishment of metabolic syndrome model by induction of fructose drinking water in male Wistar rats. BioMed Res. Int..

[85] Kampmann U., Mathiassen O.N., Christensen K.L., Buus N.H., Bjerre M., Vase H., Møller N., Kaltoft A., Poulsen P.L. (2017). Effects of Renal Denervation on Insulin Sensitivity and Inflammatory Markers in Nondiabetic Patients with Treatment-Resistant Hypertension. J. Diabetes Res..

[86] Tsioufis C., Dimitriadis K., Kasiakogias A., Kalos T., Liatakis I., Koutra E., Nikolopoulou L., Kordalis A., Ella R.O., Lau E.O. (2017). Effects of multielectrode renal denervation on elevated sympathetic nerve activity and insulin resistance in metabolic syndrome. J. Hypertens..

[87] Verloop W.L., Spiering W., Vink E.E., Beeftink M.M., Blankestijn P.J., Doevendans P.A., Voskuil M. (2015). Denervation of the renal arteries in metabolic syndrome: The DREAMS-study. Hypertension.

[88] Mulder J., Hökfelt T., Knuepfer M.M., Kopp U.C. (2013). Renal sensory and sympathetic nerves reinnervate the kidney in a similar time-dependent fashion after renal denervation in rats. Am. J. Physiol.-Regul. Integr. Comp. Physiol..

[89] DiBona G.F., Kopp U.C. (1997). Neural control of renal function. Physiol. Rev..

