# Supplementary material for: Impact of Total or Afferent Renal-Nerve Denervation on Cardiovascular and Renal Function in Sprague Dawley Rats Fed High-Fructose and High-Sodium Diet
Source: Nutrients. 2026 Sep 20;18(18):3067. doi: 10.3390/nu18183067 (PMC13610380; doi:10.3390/nu18183067)
Supplement: Supplementary file 1 [file nutrients-18-03067-s001.zip › nutrients-4545916-supplementary.pdf]

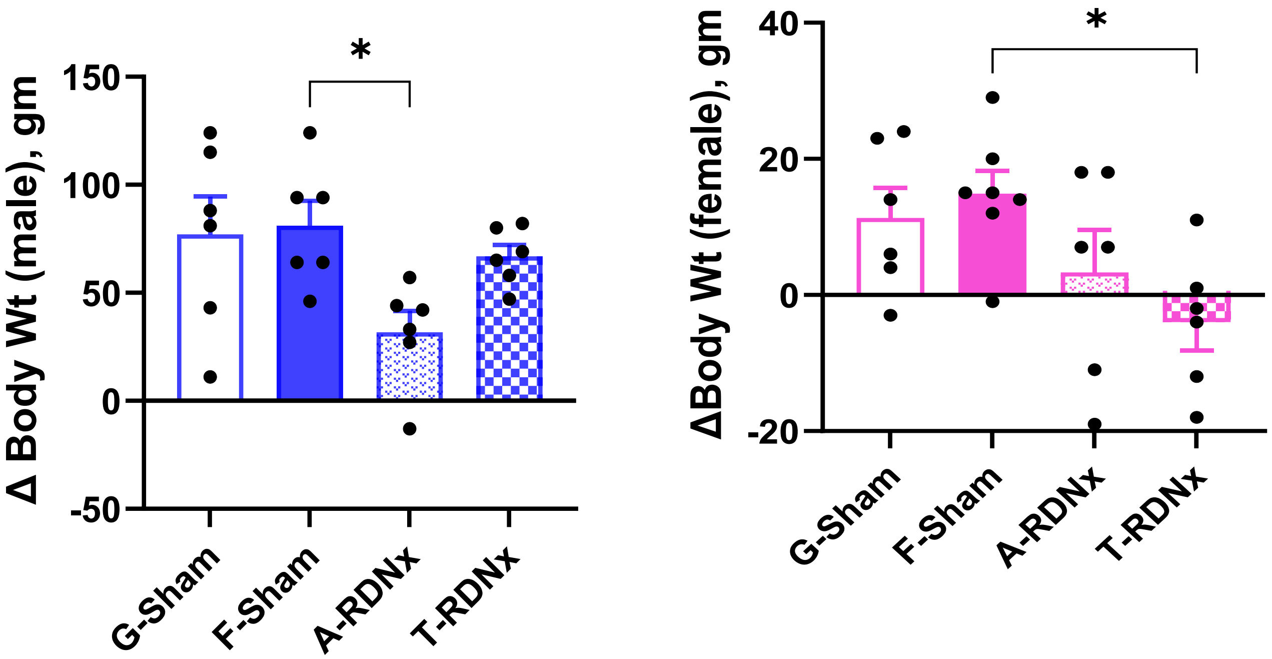

Figure S1. Change in body weight from entry into protocol until day of harvest for male and female rats. G-Sham, 20% glucose + 0.4% Na; F-Sham, 20% fructose+ 4% Na; A-RDNx, 20% fructose + 4% Na, renal deafferentation; T-RDNx, 20% fructose+ 4% Na with total renal denervation. Values are mean ± SE; * $P < 0.05$ by one-way ANOVA.

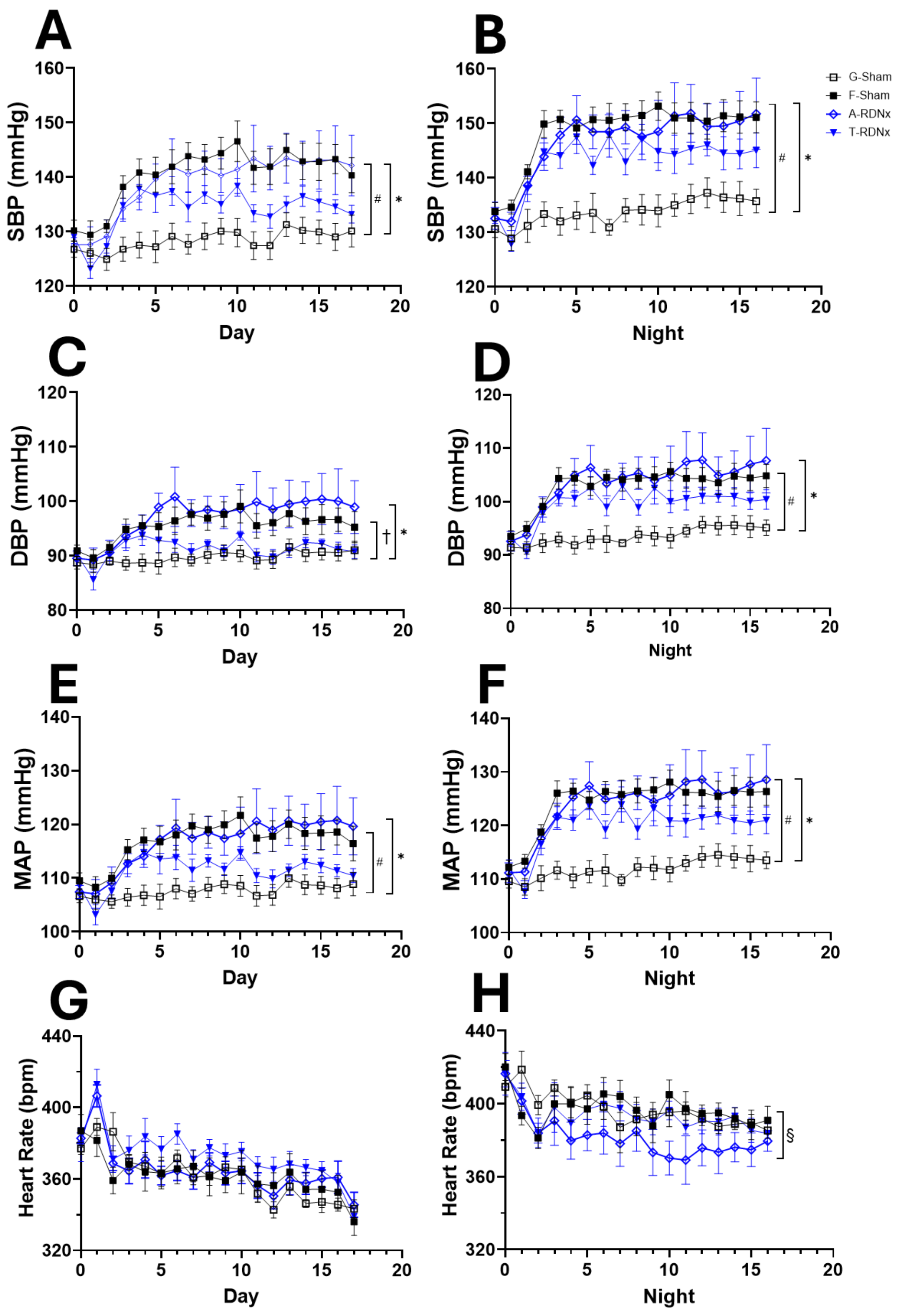

Figure S2. Systolic (SBP), diastolic (DBP), mean (MAP) blood pressures and heart rate during 12 hr-day (light cycle 0600-1800; A, C, E, G) and 12-hr night (dark cycle 1800-0600; B, D, F, H) in male rats. Group descriptions are identical to Figure S2; symbols are depicted in the legend. Values are mean ± SE. # *P* < 0.02 F-Sham vs G-Sham; † *P* < 0.05 F-Sham vs G-Sham, * *P* < 0.01 A-RDNx vs G-Sham, § *P* < 0.05 ARDNx vs G-Sham.

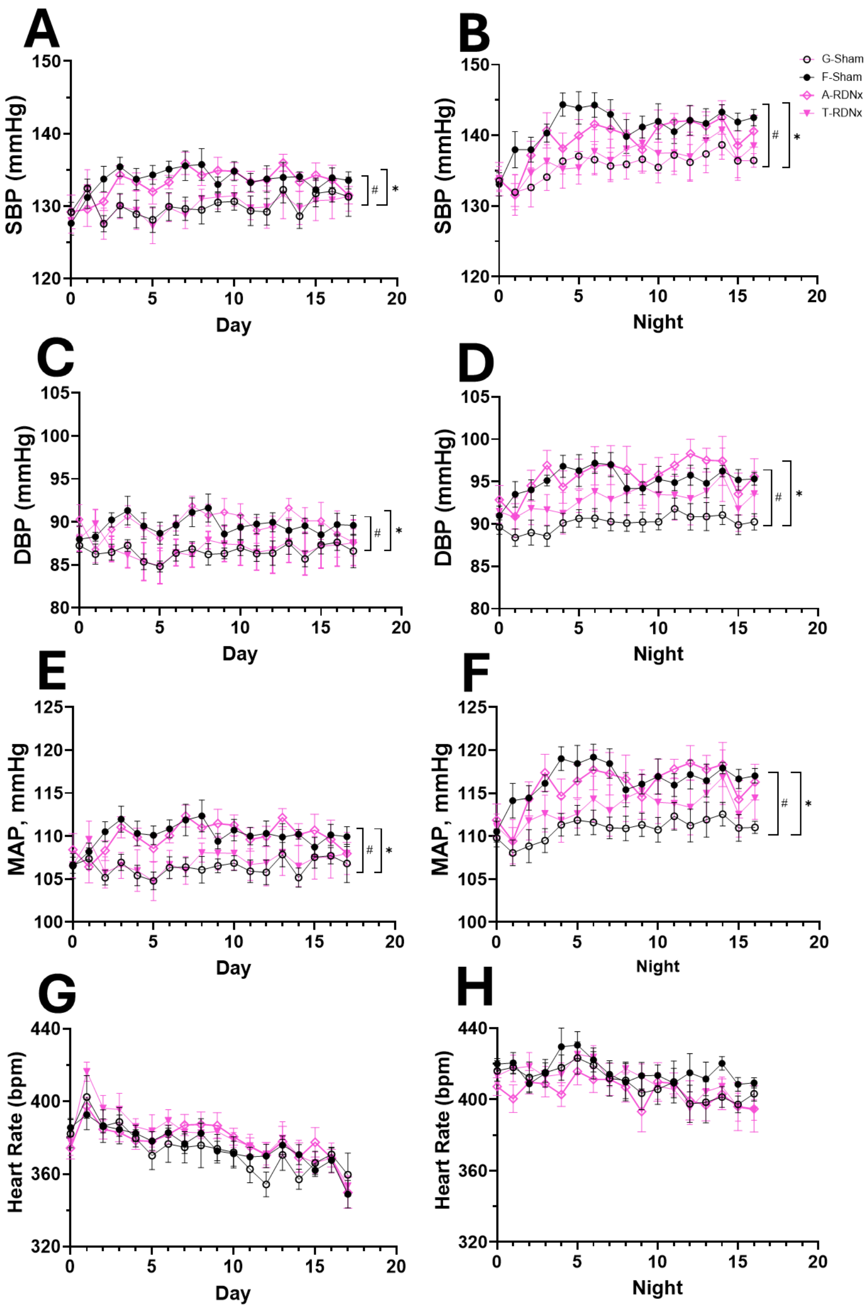

Figure S3. Systolic (SBP), diastolic (DBP), mean (MAP) blood pressures and heart rate during 12 hr-day (light cycle 0600-1800; A, C, E, G) and 12-hr night (dark cycle 1800-0600; B, D, F, H) in female rats. Group descriptions are identical to Figure S2; symbols are depicted in the legend. Values are mean ± SE. # *P* < 0.02 F-Sham vs G-Sham; † *P* < 0.05 F-Sham vs G-Sham, * *P* < 0.01 A-RDNx vs G-Sham.

<u>Systolic Blood Pressure (SBP)</u>. Supplemental Figures S2 and S3 illustrate male and female daytime and nighttime blood pressures. There was evidence of treatment effects in daytime and nighttime SBP (**Supplemental Table S3**), but circadian increases in SBP persisted with higher pressures during the active nocturnal period. The main effect of treatment indicated G-Sham had lower daytime SBP averaged across all days (estimated marginal means (EMM) = 129.19) as compared to F-Sham (EMM = 137.02, P = 0.002) and A-RDNx (EMM = 136.03, P = 0.01), but not T-RDNx (EMM = 132.01, P = 0.49); all other group comparisons were not significant. A similar pattern was observed in nighttime SBP: G-Sham (EMM = 134.33) was lower than F-Sham (EMM = 145.32, P = 0.02) and A-RDNx (EMM = 144.56, P = 0.02), but not T-RDNx (EMM = 140.84, P = 0.43), and all other treatments were similar. Multivariate tests also suggested treatments were different in the rate of change in daytime SBP over days (Day × Treatment, F (21.39, 270.93) = 1.87, P = 0.01). With a series of univariate ANOVA by day to decompose this omnibus effect, there was no evidence of the treatment effect at day 0 (F (3, 43) = 0.11, P = 0.95), and the effect of treatment was evident on day 2 (F (3, 43) = 4.37, P = 0.009) and consistently present thereafter.

<u>Diastolic Blood Pressure</u> (DBP). Diastolic blood pressure similarly showed a main effect of treatment in daytime and nighttime measurements (**Supplemental Table S3)**). The pattern of group comparisons in daytime DBP also showed G-Sham (EMM = 88.12) was lower than F-Sham (EMM = 92.55, P = 0.04) and A-RDNx (EMM = 93.76, P = 0.008), and not T-RDNx (EMM = 89.12, P = 0.92). The comparisons in nighttime measures were similar: G-Sham (EMM = 91.28) was lower than F-Sham (EMM = 99.29, P = 0.007) and A-RDNx (EMM = 101.52, P < 0.001), and not T-RDNx (EMM = 97.87, P = 0.06). All other comparisons were not significant. Treatment was similar in the rate of change in DBP across days (Day × Treatment; **Supplemental Table S3**).

<u>Heart Rate</u>. Treatments were not statistically different in daytime measures of heart rate, but did differ in average nighttime heart rate (F (3, 19) = 4.94, P = 0.01). In male rats, A-RDNx (EMM = 387.25) had a significantly lower nighttime heart rate than G-Sham (EMM = 406.59, P = 0.035), F-Sham (EMM = 409.77, P = 0.004) and T-RDNx (EMM = 410.40, P = 0.007); all other comparisons were not significant in male rats (**Figure S2)**. No differences in heart rate were observed in the female rat groups (**Figure S3)**. Treatment was otherwise similar in the rate of change in heart rate over observation days (Day × Treatment; **Supplemental Table S3**).

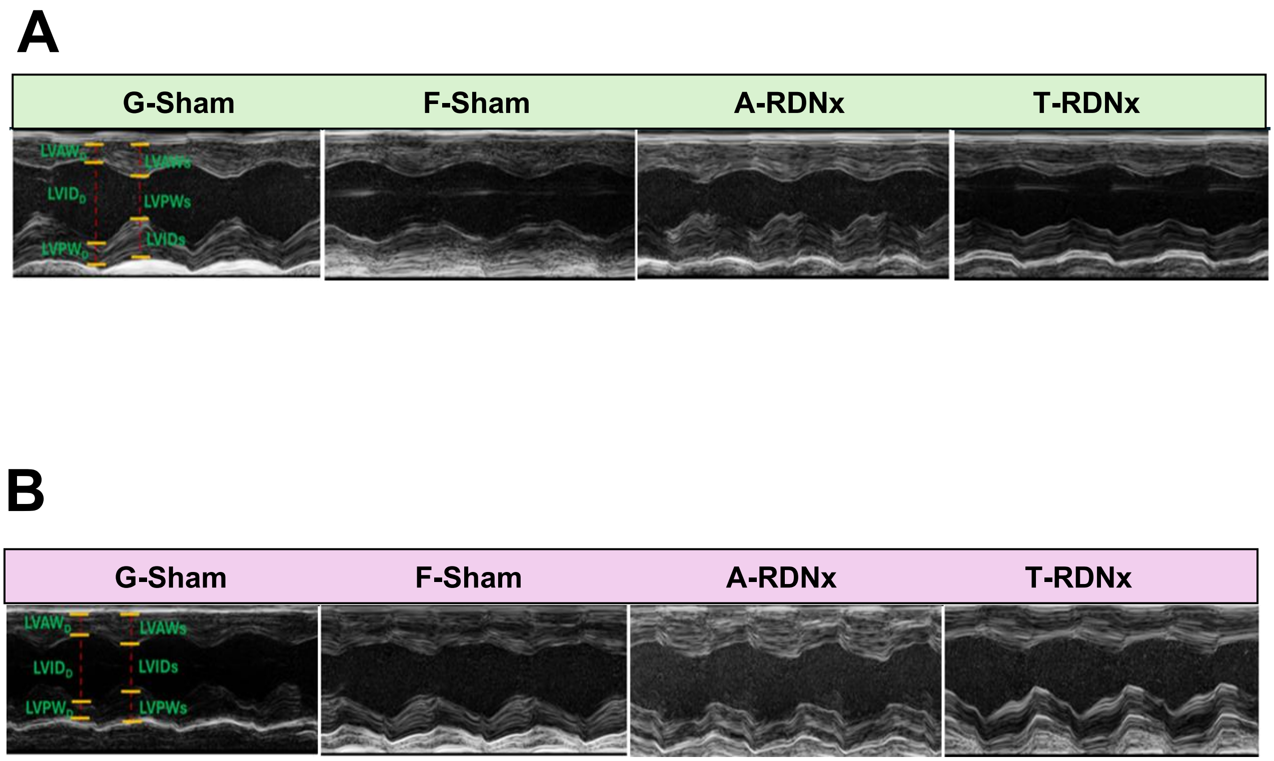

Figure S4. Representative M-mode echocardiogram images in male (A) and female (B) rats from each of the groups. $LVID_s$, left ventricular systolic internal diameter; $LVID_D$, left ventricular diastolic internal diameter; LVAW, left ventricular anterior wall width (s, systole; d, diastole); LVPW, left ventricular posterior wall width.

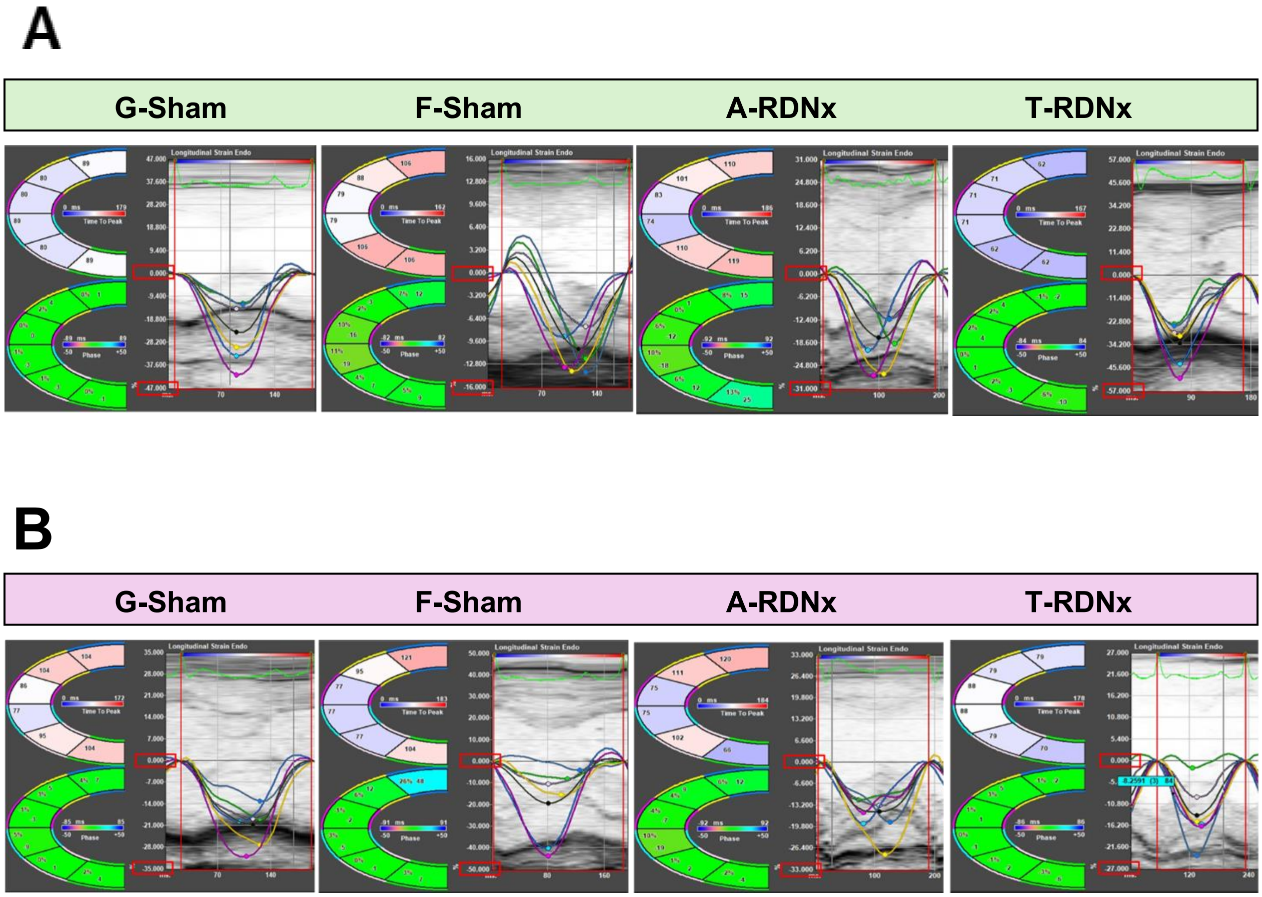

Figure S5. Representative strain measurements for male (A) and female (B) groups assessed by speckle tracking echocardiography.

Supplementary Table S1: TestDiet 20% Glucose, 1.1% K, 0.4% Na

**Protein, %**	18.6	**Minerals**	
Arginine,%	0.73	Calcium,%	0.6
Histidine,%	0.54	Phosphorus, %	0.56
Isoleucine,%	1	Potassium, %	1.22
Leucine,%	1.82	Magnesium, %	0.07
Lysine,%	1.53	Sodium, %	0.4
Methionine,%	0.69	Chloride, %	1.3
Cystine, %	0.08	Fluorine, ppm	5
Phenylalanine, %	1	Iron, ppm	68
Tyrosine,%	1.06	Zinc, ppm	27
Threonine, %	0.81	Manganese, ppm	65
Tryptophan, %	0.23	Copper, ppm	15
Valine, %	1.2	Cobalt, ppm	3.2
Alanine,%	0.58	Iodine, ppm	0.57
Aspartic Acid, %	1.35	Chromium, ppm	3
Glutamic Acid, %	4.29	Molybdenum, ppm	0.82
Glycine, %	0.41	Selenium, ppm	0.3
Proline, %	2047		
Serine, %	1.16	**Vitamins**	
Taurine, %	0	Vitamin A, IU/g	22.1
		Vitamin D-3, IU/g	2.2
**Fat, %**	**10**	Vitamin E, IU/kg	55.2
Cholesterol, ppm	48	Vitamin K, ppm	10.4
Linoleic Acid, %	3.34	Thiamin, ppm	18.8
Linolenic Acid, %	0.07	Riboflavin, ppm	20.7
Archidonic Acid, %	0.01	Niacin, ppm	90
Omega-3 Fatty Acids, %	0.07	Pantothenic Acid, ppm	56
Total Saturated Fatty Acid, %	2.72	Folic Acid, ppm	4.2
Total Monosaturated Fatty Acid, %	3.31	Pyridoxine, ppm	16.5
Polyunsaturated fatty Acid, %	3.42	Biotin, ppm	0.4
**Fiber(max), %**	**4.6**	Vitamin B-12, mcg/kg	23
**Carbohydrates, %**	**58.3**	Choline Chloride, ppm	1400
**Energy (kcal/g)**	**3.98**	Ascorbic Acid, ppm	0

Supplementary Table S2: TestDiet 20% Fructose, 1.1% K, 4.0% Na

**Protein, %**	18.6	**Minerals**	
Arginine,%	0.73	Calcium,%	0.6
Histidine,%	0.54	Phosphorus, %	0.56
Isoleucine,%	1	Potassium, %	1.22
Leucine,%	1.82	Magnesium, %	0.07
Lysine,%	1.53	Sodium, %	4
Methionine,%	0.69	Chloride, %	6.85
Cystine, %	0.08	Fluorine, ppm	5
Phenylalanine, %	1	Iron, ppm	68
Tyrosine,%	1.06	Zinc, ppm	27
Threonine, %	0.81	Manganese, ppm	65
Tryptophan, %	0.23	Copper, ppm	15
Valine, %	1.2	Cobalt, ppm	3.2
Alanine,%	0.58	Iodine, ppm	0.57
Aspartic Acid, %	1.35	Chromium, ppm	3
Glutamic Acid, %	4.29	Molybdenum, ppm	0.82
Glycine, %	0.41	Selenium, ppm	0.3
Proline, %	2047		
Serine, %	1.16	**Vitamins**	
Taurine, %	0	Vitamin A, IU/g	22.1
		Vitamin D-3, IU/g	2.2
**Fat, %**	10	Vitamin E, IU/kg	55.2
Cholesterol, ppm	48	Vitamin K, ppm	10.4
Linoleic Acid, %	3.34	Thiamin, ppm	18.8
Linolenic Acid, %	0.07	Riboflavin, ppm	20.7
Archidonic Acid, %	0.01	Niacin, ppm	90
Omega-3 Fatty Acids, %	0.07	Pantothenic Acid, ppm	56
Total Saturated Fatty Acid, %	2.72	Folic Acid, ppm	4.2
Total Monosaturated Fatty Acid, %	3.31	Pyridoxine, ppm	16.5
Polyunsaturated fatty Acid, %	3.42	Biotin, ppm	0.4
**Fiber(max), %**	4.6	Vitamin B-12, mcg/kg	23
**Carbohydrates, %**	49.1	Choline Chloride, ppm	1400
**Energy (kcal/g)**	3.61	Ascorbic Acid, ppm	0

Supplementary Table S3: Summary of Repeated Measures ANOVA Results

	Outcome Assessment					
	Daytime			Nighttime		
Predictor	Systolic Blood Pressure	Diastolic Blood Pressure	Heart Rate	Systolic Blood Pressure	Diastolic Blood Pressure	Heart Rate
**Within-Subject Effects**						
**Day**	**18.35, *P* < 0.001**	**5.49, *P* < 0.001**	**38.52, *P* < 0.001**	**14.23, *P* < 0.001**	**7.28, *P* < 0.001**	**7.55, *P* < 0.001**
**Day × Sex**	**7.22, *P* < 0.001**	**4.19, *P* = 0.001**	1.50, *P* = 0.14	2.62, *P* = 0.04	2.06, *P* = 0.11	1.66, *P* = 0.13
**Day × Treatment**	**1.87, *P* = 0.01**	4.19, *P* = 0.05	1.46, *P* = 0.07	0.86, *P* = 0.59	0.62, *P* = 0.78	1.39, *P* = 0.14
**Day × Sex × Treatment**	1.33, *P* = 0.15	1.16, *P* = 0.30	0.82, *P* = 0.73	0.80, *P* = 0.66	0.62, *P* = 0.79	0.86, *P* = 0.64
**Average Effect**						
**Sex**	5.85, *P* = 0.02	**19.74, *P* < 0.001**	**11.15, *P* < 0.01**	1.68, *P* = 0.21	**8.73, *P* = 0.01**	6.77, *P* = 0.02
**Treatment**	**6.67, *P* < 0.001**	**5.43, *P* = 0.003**	1.41, *P* = 0.26	**4.69, *P* = 0.01**	**5.20, *P* = 0.01**	**4.94, *P* = 0.01**
**Sex × Treatment**	1.71, *P* = 0.18	0.52, *P* = 0.67	0.16, *P* = 0.92	1.46, *P* = 0.26	0.53, *P* = 0.67	1.22, *P* = 0.33

Note: **Omnibus F-tests are reported with significance testing**. Multivariate effects of the within-subject predictors are reported with Greenhouse-Geiser adjustment. Effects that were significant following familywise error adjustment are bolded (α’ = 0.01).

Sex Differences. Independent of treatment, average sex differences were evident in diastolic blood pressure in daytime ($F(1,38) = 19.74$, $P < 0.001$) and nighttime ($F(1,19) = 8.73$, $P = 0.008$), both indicating females had average lower blood pressure (EMM female daytime = 139.90 vs. male daytime = 142.62; female nighttime = 94.98 vs. male nighttime = 100.00); and in day time heart rate ($F(1,38) = 11.15$, $P = 0.002$), indicating females had higher heart rate (EMM female = 376.76 vs. male = 364.65). There was additional evidence of sex differences in the rate of change in daytime SBP ($F(7.13, 270.93) = 1.87$, $P = 0.01$) and DBP ($F(5.09, 193.54) = 4.19$, $P = 0.001$). The descriptive analysis indicated male and female animals were similar on day 0, and beginning day 1 female animals demonstrated a sustained, slightly elevated SBP and slight decreasing DBP across all observation days, whereas males had greater variability across days that resulted a different change slope over time. Despite an increase in SBP in both male and female rats, males demonstrated a greater increase in the fructose high sodium group compared with female rats: 18.2 ± 3.6 vs 4.9 ± 1.2 mmHg ($P < 0.01$). Likewise, the rise in MAP was greater in male vs female rats: 14.3 ± 2.9 vs 4.7 ± 1.5 mmHg ($P < 0.02$), but the change in DBP was not statistically different between the sexes: 10.3 ± 1.8 vs 3.8 ± 1.8 mmHg (P = 0.054). Taken together, females had greater consistency in daytime blood pressure than males over the observation period, with rank order lower average DBP and higher daytime heart rate than males. However, the effect of treatment was comparable between sexes.
